# Cellular and microenvironmental cues that promote macrophage fusion and foreign body response

**DOI:** 10.3389/fimmu.2024.1411872

**Published:** 2024-07-05

**Authors:** Chloe L. Stewart, Andrew L. Hook, Mischa Zelzer, Maria Marlow, Anna M. Piccinini

**Affiliations:** ^1^ School of Pharmacy, University of Nottingham, Nottingham, United Kingdom; ^2^ Institute of Developmental and Regenerative Medicine, University of Oxford, Oxford, United Kingdom

**Keywords:** macrophage, macrophage fusion, foreign body response, foreign body giant cell, Toll-like receptor signalling, fibroblast

## Abstract

During the foreign body response (FBR), macrophages fuse to form foreign body giant cells (FBGCs). Modulation of FBGC formation can prevent biomaterial degradation and loss of therapeutic efficacy. However, the microenvironmental cues that dictate FBGC formation are poorly understood with conflicting reports. Here, we identified molecular and cellular factors involved in driving FBGC formation *in vitro*. Macrophages demonstrated distinct fusion competencies dependent on monocyte differentiation. The transition from a proinflammatory to a reparative microenvironment, characterised by specific cytokine and growth factor programmes, accompanied FBGC formation. Toll-like receptor signalling licensed the formation of FBGCs containing more than 10 nuclei but was not essential for cell-cell fusion to occur. Moreover, the fibroblast-macrophage crosstalk influenced FBGC development, with the fibroblast secretome inducing macrophages to secrete more PDGF, which enhanced large FBGC formation. These findings advance our understanding as to how a specific and timely combination of cellular and microenvironmental factors is required for an effective FBR, with monocyte differentiation and fibroblasts being key players.

## Introduction

Macrophages choreograph the innate immune response by detecting microenvironmental changes, phagocytosing pathogens, releasing soluble mediators to direct cell responses, and regulating tissue repair (1). However, a distinctive phenomenon of macrophages is their unique ability to fuse into multinucleated giant cells (MGCs) that perform highly specialised functions depending on their anatomical location and fusion stimulus (2). Specifically, macrophage-derived MGCs include osteoclasts, which resorb bone and regulate bone tissue homeostasis (3), Langhans giant cells (LGCs), which arise in response to granulomas induced by microorganisms like *Mycobacterium tuberculosis* or non-infectious granulomatous disorders (4), and foreign body giant cells (FBGCs), which are generated exclusively during the immune response to implanted biomaterials (5). MGC formation results from ‘frustrated phagocytosis’, which occurs when individual macrophages cannot phagocytose large cell aggregates or foreign materials (6). Consequently, MGCs release tissue-degrading molecules such as matrix metalloproteinases (MMPs), acids and reactive oxygen species to destroy non-phagocytosable substrates (7). Whilst MGCs provide biological benefits to the host such as restricting the spread of microorganisms (8), FBGC formation can hinder the therapeutic efficacy of biomaterials (9, 10).

The presence of FBGCs is a hallmark of the foreign body response (FBR) that distinguishes it from chronic inflammation (11) and a critical determinant of the longevity of implanted biomaterials. FBGCs can persist at the material-tissue interface for the lifetime of the implant (12) where they induce severe biomaterial degradation by creating an oxidative environment (13). Conversely, FBGC formation also coincides with a transition of the microenvironment to one that favours tissue repair and extracellular matrix (ECM) remodeling (14). Macrophages actively contribute to wound healing by synthesising collagen (15) and, in conjunction with FBGCs, secrete growth factors that stimulate fibroblasts to synthesise new ECM (14). However, the persistence of FBGCs can lead to prolonged fibroblast activation and excessive collagen deposition, thus contributing to the fibrotic encapsulation of foreign biomaterials (16). In addition to inducing tissue repair activities, the production of MMPs by macrophages and FBGCs also mediates the resorption and remodelling of the deposited fibrous tissue (17). Consequently, macrophage depletion results in the formation of an irreversible acellular fibrotic capsule around the biomaterial that can impede the efficacy of drug delivery systems or biochemical monitoring sensors (18).

Macrophages and FBGCs have a demonstrable role in directing the progression of the FBR (14, 16). It is hypothesised that modulating macrophages and FBGC formation may control the FBR in a desirable manner (10, 19). However, macrophage multinucleation is a dynamic and multistage process in which a myriad of microenvironmental factors programme macrophages to participate in fusion (4, 20). During the FBR, these microenvironmental cues may include inflammatory signals (e.g. anaphylatoxins, microbial products, danger-associated molecular patterns), soluble factors (e.g. cytokines, chemokines, growth factors), cells specific to the tissue type and anatomical location (e.g. fibroblasts), and the implanted biomaterial. Over the past three decades, research focused on the *in vitro* formation of FBGCs (21) and the impact of biomaterial properties on specific elements of the FBR (22, 23) has elucidated critical signalling pathways and molecular mediators of macrophage fusion, including STAT6 (24, 25), DC-STAMP (26), and integrins (27, 28). However, the role of microenvironmental cues on FBGC formation at the cellular and tissue level remains largely underinvestigated. To address this, we performed a holistic evaluation of relevant molecular and cellular interactions and characterised their effect on FBGC formation. Building upon the established understanding that macrophages require a permissive substrate and STAT6-activating cytokines to induce fusion (20), we identified that monocyte differentiation and macrophage phenotype were determinants of macrophage fusion competency and FBGC formation. We also demonstrated that toll-like receptor (TLR) activation was a driver of large FBGC formation, and fusogenic conditions were associated with a highly inflammatory microenvironment for the first 7 days. Over time, the fusion process coincided with a transition of the microenvironment to one that favoured tissue repair activities, and exogenously added growth factors further enhanced FBGC formation. Finally, we identified fibroblasts as key cells in mediating FBGC development, with fibroblast-secreted soluble mediators enhancing FBGC formation and, strikingly, macrophage-fibroblast direct contact enabling macrophage fusion on non-permissive substrates. Understanding major drivers of FBGC formation may identify new avenues for targeting FBGC development to direct the FBR in a favourable manner.

## Materials and methods

### Isolation and culture of primary human monocytes

Peripheral blood mononuclear cells (PBMCs) were extracted from peripheral blood (NHS Blood & Transplant) using Lymphoprep density gradient medium (Stemcell Technologies) in SepMate PBMC Isolation Tubes (Stemcell Technologies) followed by CD14^+^ monocyte isolation with human CD14 MicroBeads (Miltenyi Biotec). Isolated monocytes were cultured in RPMI 1640 containing 10% (v/v) FBS (Gibco) and penicillin/streptomycin (100 U/100 µg/mL; Lonza). Monocytes were differentiated into macrophages by supplementing media with recombinant human macrophage-colony stimulating factor (M-CSF) (25–100 ng/mL; PeproTech) or granulocyte macrophage-colony stimulating factor (GM-CSF) (2.5–10 ng/mL; PeproTech). Macrophages were cultured for up to 28 days with media replacement every three to four days.

### Inducing macrophage fusion and FBGC formation

Polyethylene terephthalate (PET) film (0.1 mm thickness; Goodfellow Cambridge) was sterilised in 70% (v/v) ethanol for 30 minutes followed by three washes in PBS. Sterilised PET films were transferred to 96-well plates (Corning) and incubated under UV light (254 nm) for 20 minutes. Monocytes (1.5x10^5^ cells/well) were seeded onto PET surfaces and incubated in M-CSF- or GM-CSF-supplemented media for 72 hours, unless otherwise stated. Thereafter, media was replaced with fresh media containing M-CSF or GM-CSF and either IL-4 (10 ng/mL; PeproTech), IL-13 (10 ng/mL; PeproTech) or both IL-4 (5 ng/mL) and IL-13 (5 ng/mL). Each experimental condition was repeated in triplicate and cultured for up to 28 days with media replacement every three to four days.

### Cytology of macrophages and FBGCs

Cultures were terminated by fixing cells in 50 µL/well of ice-cold methanol for five minutes. Macrophages and FBGCs were stained with 50 µL/well of May-Grünwald (Sigma-Aldrich) for one minute and washed with 200 µL/well of PBS, followed by staining with 50 µL/well of Giemsa (Sigma-Aldrich) for five minutes and washing with deionised water twice (29). Plates were air-dried overnight prior to imaging.

Five sampling points were imaged per well (Zeiss Axioplan) and processed using Fiji (30). The quantitative data obtained from each of the five images was totalled to represent the entire well. Each experimental condition was repeated in triplicate with quantitative data reported as average with standard deviation. Experimental observations were confirmed with monocyte-derived macrophages (MDMs) obtained from multiple biological donors and data for statistical analyses and figure generation reported as average of the means ± standard error of the mean (SEM).

Multinucleated cells and FBGCs were defined as a cell that contained ≥2 nuclei/cell and ≥4 nuclei/cell, respectively. Nuclei counting and measurement of cell area and circularity was performed manually using Fiji. Example images of counting individual macrophages and FBGCs are shown in **Supplementary Figure S8**. Macrophage fusion was calculated according to **Equation 1**.


(1) $$\text{Macrophage fusion }\left(\%\right)=\frac{\text{Nmc}}{\text{N}}\times \;100\text{Nmc = Number of nuclei counted within multinucleated cells}\text{N = Number of nuclei counted in total}$$

### Adhesion assay

Monocytes were seeded on culture surfaces as above and left to adhere for one hour at 37°C. Cells were fixed in 4% (v/v) PFA in PBS for 15 minutes at room temperature (RT) and stained with 1% (w/v) toluidine blue O in 4% PFA for 10 minutes at RT. Cells were washed thoroughly with PBS to remove excess dye followed by rinsing with deionised water prior to imaging (Zeiss Axioplan). Stained monocytes were lysed in 2% (w/v) sodium dodecyl sulphate until a uniform colour was achieved. The absorbance at 590 nm was measured using a microplate reader (BioTek Synergy HT). Each biological donor was repeated in quadruplicate.

### Enzyme-linked immunosorbent assay

Cell supernatants were analysed with ELISA kits to quantify TNF-α, IL-1β, IL-6, IL-8, IL-10, CCL2, PDGF-BB and TGF-β1 (Human DuoSet; R&D Systems), and tenascin-C (Abcam), according to the manufacturer’s instructions. Absorbance at 450 nm was read on a microplate reader (BioTek Synergy HT) and the data analysed in GraphPad Prism (version 9.4.1). Each biological donor was repeated in duplicate.

### TLR4 inhibition

TAK-242 (1 µg/mL (15); CLI-095, InvivoGen) was added to monocytes in conjunction with IL-4 on day 3 of culture. Dimethyl sulfoxide (DMSO) was used as solvent for TAK-242 and added to monocytes in the control condition. Each biological donor was repeated in triplicate.

### TLR activation

Heat-killed *Staphylococcus aureus* (HKSA; 1x10^7^ cells/mL; InvivoGen) was added to monocytes during initial cell seeding. Macrophage differentiation and fusion was induced as described above. The experiment was terminated after 21 days of culture and macrophages stained with Giemsa for imaging. Each biological donor was repeated in triplicate.

### FBGC and fibroblast co-culture

Human primary monocytes, obtained from at least three biological donors, and human foreskin fibroblasts (BJ; CRL-2522, ATCC) were cultured in Dulbecco’s modified Eagle’s medium (DMEM) containing 10% (v/v) FBS and penicillin/streptomycin (100 U/100 µg/mL). Cultures were continued for at least three weeks, unless stated otherwise, with media replaced with fresh every three to four days.

#### Indirect co-culture

Fibroblasts (9x10^4^ cells/well) were seeded onto glass coverslips in 24-well plates and left to adhere for 24–72 hours. Monocytes (3x10^5^ cells/insert) were seeded into Transwell inserts (polyethylene terephthalate; 0.4 µm pore diameter; Corning). Only media within the inserts was supplemented with M-CSF (50 ng/mL) and IL-4 (10 ng/mL) to induce macrophage differentiation and fusion. Each biological donor was repeated in duplicate.

#### Conditioned media

Monocytes (1.5x10^5^ cells/well) were seeded onto TCPS or PET surfaces in 96-well plates and incubated in media containing M-CSF (50 ng/mL). Fibroblasts (2x10^4^ cells/well) were seeded in 12-well plates without cytokines. After 72 hours, fibroblast supernatants were collected, diluted with an equal volume of fresh media and added to monocyte cultures. Fibroblast-conditioned media was also supplemented with M-CSF (50 ng/mL) and IL-4 (10 ng/mL) where indicated. Cultures were continued for 21 days with media replacement every two to three days. Each biological donor was repeated in triplicate.

#### Direct co-culture

Fibroblasts (5x10^4^ cells/well) were seeded in 96-well plates and left to adhere for 24 hours to form a semi-confluent layer. Monocytes (1.5x10^5^ cells/well) were seeded on fibroblast cultures or TCPS surfaces and incubated with M-CSF and IL-4 as above. Each biological donor was repeated in triplicate.

### Platelet-derived growth factor

Monocytes were seeded on PET film and incubated in a cytokine cocktail of M-CSF and IL-4 for seven days. Recombinant human PDGF-BB (PeproTech) was added from day 7 at increasing concentrations (day 7: 100 pg/mL, days 10 and 14: 200 pg/mL, day 17: 400 pg/mL) to mimic an increase in PDGF secretion observed by fusion-competent macrophages and FBGCs over time. Cultures were terminated for image analysis after 21 days of culture. Each biological donor was repeated in triplicate.

### Statistical analysis

Statistical analyses were performed using two-way ANOVA followed by the Šídák or Tukey multiple comparison tests or unpaired t-tests with GraphPad Prism (version 9.4.1). P values are reported as * p<0.05, ** *p*<0.01, *** *p*<0.005, **** *p*<0.001.

## Results

### M-CSF and GM-CSF have distinct effects on macrophage fusion

**Figure 1:**
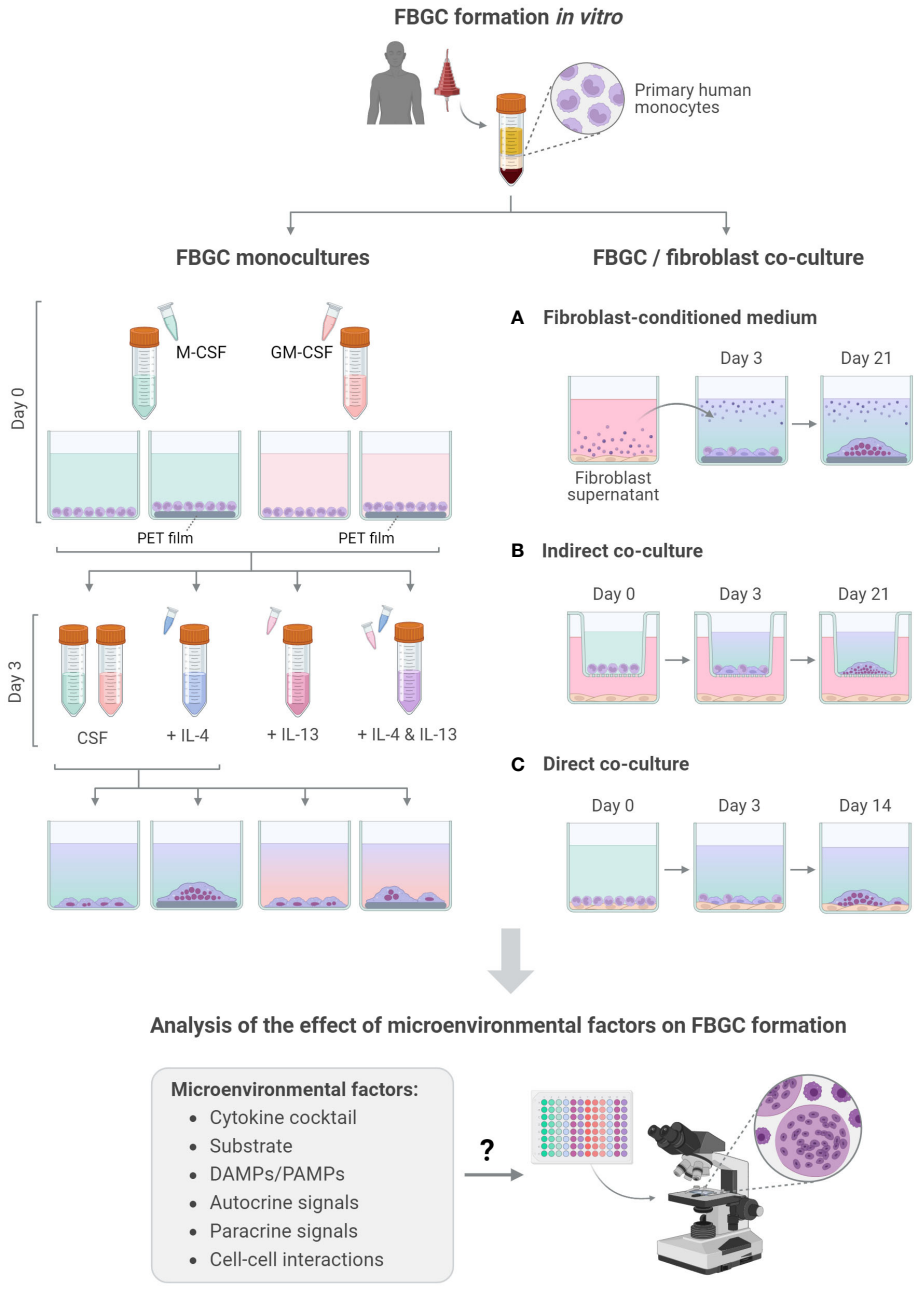
Overview of FBGC formation and analysis. Schematic of the experimental workflow used to induce the fusion of primary human monocyte-derived macrophages into FBGCs *in vitro* and investigate the effect of molecular **(A, B)** and cellular **(C)** factors on FBGC formation. Created with BioRender.com.

**Figure 2:**
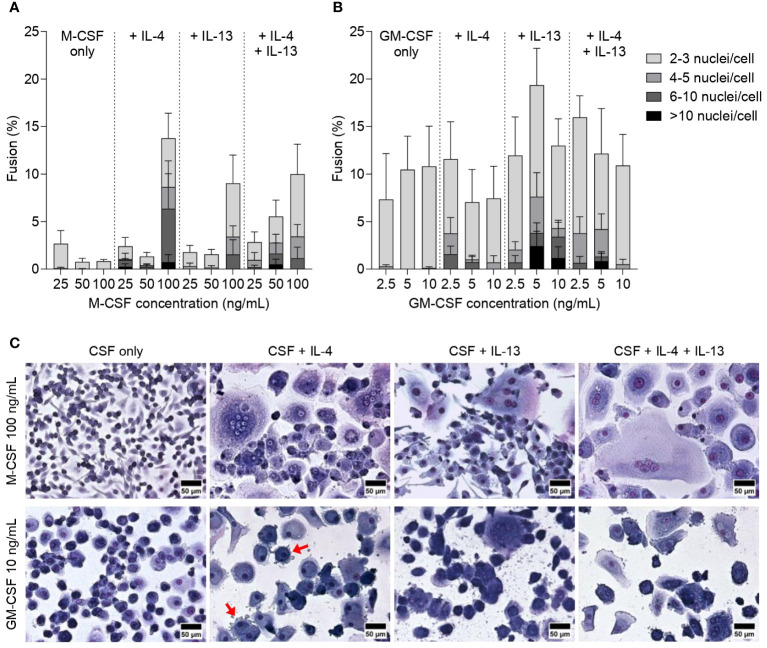
Effect of M-CSF and GM-CSF on FBGC formation. Percentage of macrophage fusion in response to **(A)** M-CSF (25–100 ng/mL) and **(B)** GM-CSF (2.5–10 ng/mL) with stacked bars showing the percentage of nuclei within FBGC containing a total of 2 to 3, 4 to 5, 6 to 10 and/or >10 nuclei/cell. Macrophages were cultured in the absence or presence of IL-4 (10 ng/mL), IL-13 (10 ng/mL) or both IL-4 and IL-13 (5 ng/mL each) for 28 days. Data are means ± SEM of three donors. **(C)** Macrophages and FBGCs were stained with May-Grünwald and Giemsa. Images are representative of three independent experiments, each with a different donor. Scale bar, 50 μm. Red arrows indicate dendritic cell morphologies.

Macrophage-colony stimulating factor (M-CSF) and granulocyte macrophage-colony stimulating factor (GM-CSF) are hematopoietic growth factors that induce monocyte differentiation and generate mature, but phenotypically distinct (31) myeloid cell populations *in vitro* (32, 33). M-CSF is expressed at biologically active concentrations in the circulation under homeostatic conditions and produces mature macrophages of a neutral, non-biased phenotype (32). Conversely, GM-CSF is expressed during tissue damage or infection and is a driver of tissue inflammation, polarising macrophages towards a pro-inflammatory phenotype (33). However, GM-CSF has also been widely used *in vitro* to obtain dendritic cells (34). Previous studies have utilised either GM-CSF (24, 25, 35) or M-CSF (2, 36) to induce monocyte differentiation prior to FBGC formation without consideration of their potential impact on macrophage fusion. To test whether macrophage phenotype influences FBGC formation, we differentiated monocytes with M-CSF (M-MDMs) or GM-CSF (GM-MDMs) to represent macrophages within homeostatic and inflammatory environments, respectively (32) (**Figure 1**), and classified multinucleated cells containing ≥4 nuclei/cell as FBGCs (21). Both M-MDMs and GM-MDMs exhibited low levels of fusion (**Supplementary Figures S1A, B**), with many multinucleated cells being not true FBGCs (**Figures 2A, B**). However, M-MDMs and GM-MDMs displayed different cell morphologies (**Figure 2C**) wherein M-MDMs were a mixed population of small round and elongated cells whilst GM-MDMs possessed large and round morphologies, which indicated alternatively-activated and pro-inflammatory phenotypes (37), respectively.

Interleukins IL-4 and IL-13 are vital cytokines to induce macrophage fusion and large FBGCs *in vitro* (24, 25) and *in vivo* (38). IL-4 and IL-13 both activate the signal transducer and activator of transcription factor 6 (STAT6) pathway (39) that enables macrophages to adopt a fusion-competent state (20). Most studies utilised IL-4 to induce macrophage fusion but there is limited evidence to indicate whether IL-4 or IL-13 have different effects on FBGC formation. To address this gap in knowledge, we added interleukins individually or in combination 72 hours following monocyte seeding as per previous reports (20, 24) and analysed FBGC formation (**Figure 1**). The addition of interleukins did not significantly affect macrophage fusion by either M-MDMs or GM-MDMs (**Supplementary Figures S1A, B**). However, macrophages differentiated with 100 ng/mL of M-CSF had a greater percentage of fusion (**Figure 2A**, **Supplementary Figure S1A**) and number of fused macrophages in FBGCs (**Supplementary Figure S1C**) following interleukin treatment, especially when IL-4 was added, compared to M-MDMs differentiated with lower concentrations of M-CSF. Conversely, GM-MDMs differentiated with 5 ng/mL or 10 ng/mL of GM-CSF exhibited a slight increase in fusion (**Figure 2B**, **Supplementary Figure S1B**) and number of fused macrophages in FBGCs (*p*<0.05, **Supplementary Figure S1D**) in response to IL-13 polarisation. Whilst characteristic macrophage morphologies were observed in most cytokine combinations, GM-MDMs polarised by IL-4 produced cell morphologies resemblant of dendritic cells (**Figure 2C**) and during the 28 days of culture their cell density significantly decreased (data not shown).

Together, these data demonstrated that M-MDMs and GM-MDMs respond differently to IL-4 and IL-13, with M-MDMs being more responsive to IL-4 and GM-MDMs to IL-13. These observations suggest that macrophage differentiation and polarisation shape FBGC formation.

### The combination of a foreign biomaterial with IL-4 licenses FBGC formation

**Figure 3:**
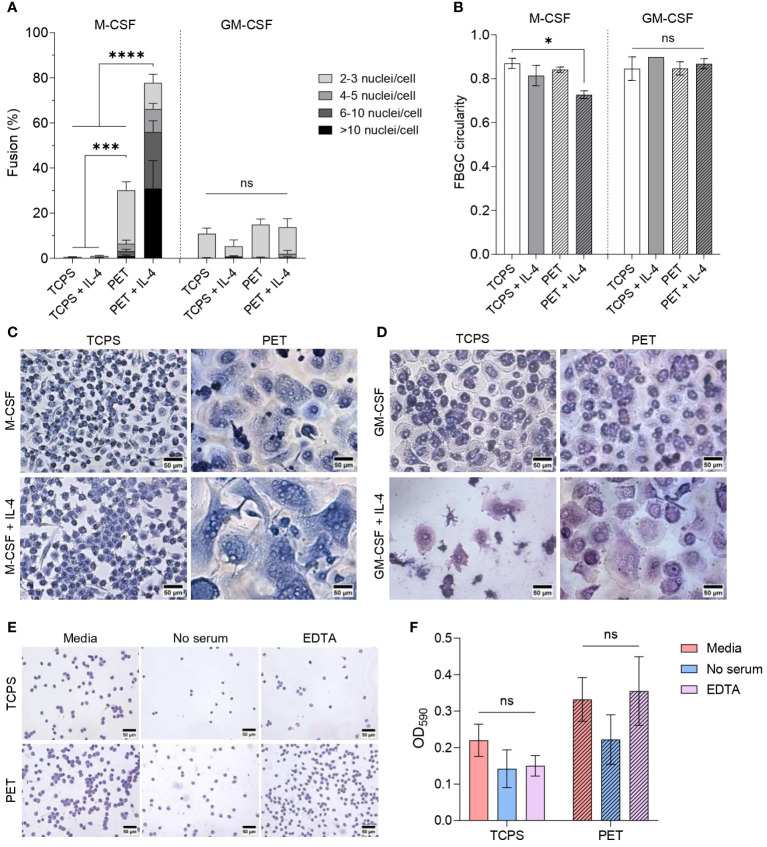
Effect of cytokine combination and substrate surface on FBGC formation. **(A)** Percentage of macrophage fusion in response to culturing macrophages on PET following differentiation with M-CSF (50 ng/mL) or GM-CSF (5 ng/mL) and polarisation with IL-4 (10 ng/mL). Stacked bars show the percentage of nuclei within FBGC containing a total of 2 to 3, 4 to 5, 6 to 10 and/or >10 nuclei/cell. Data is the mean ± SEM of four (GM-CSF) and five (M-CSF) biological donors. Statistical significance was determined by two-way ANOVA with the Tukey multiple comparison test (*p<0.05, ***p<0.005, ****p<0.001). **(B)** Average circularity of FBGCs formed in different culture conditions. Data is the mean ± SEM of three (GM-CSF) and four (M-CSF) biological donors. Statistical significance was determined by two-way ANOVA with the Tukey multiple comparison test (* *p*<0.05). Representative images of **(C)** M-CSF- and **(D)** GM-CSF-differentiated macrophages and FBGCs cultured for 28 days on TCPS or PET without or with IL-4. Cells stained with May-Grünwald and Giemsa prior to imaging. Scale bar, 50 μm. **(E)** Representative images of toluidine blue-stained monocytes adhered to TCPS and PET substrates one hour after seeding in regular culture media, media without serum, and media with EDTA. Scale bar, 50 μm. **(F)** Absorbance of toluidine blue at 590 nm following monocyte lysis to represent the proportion of monocytes adhered to TCPS and PET substrates after one hour when cultured in regular media, media without serum, and media with EDTA. Data is the mean ± SEM of three biological donors. No statistical significance (ns) was determined by two-way ANOVA with the Šídák multiple comparison test.

Despite reports of interleukins inducing large FBGC formation on standard tissue-culture surfaces (5, 20, 24, 25), our experiments yielded low levels (<20%) of fusion when macrophages were cultured on tissue culture polystyrene (TCPS). To explore whether macrophage fusion is enhanced in the presence of a biomaterial with a known foreign body reaction, we seeded monocytes on polyethylene terephthalate (PET) film, which has previously induced FBGC formation *in vivo* (40) and *in vitro* (41). Changing the culture substrate from TCPS to PET induced a striking enhancement (*p*<0.005) in the fusion of M-MDMs (**Figure 3A**). M-MDMs on PET also adopted large, round morphologies that contrasted the small, elongated M-MDMs on TCPS (**Figure 3C**). However, culturing GM-MDMs on PET did not induce any statistically significant increase in macrophage fusion compared to GM-MDMs on TCPS (**Figure 3A**), nor any observable changes in cell morphologies (**Figure 3D**).

We next determined the combined effect of PET and interleukins on fusion. Adding IL-4 to M-MDMs on PET remarkably enhanced (*p*<0.001) macrophage fusion and the formation of large (>10 nuclei/cell) FBGCs (**Figure 3A**). Large FBGC formation was associated with reduced cell circularity (**Figure 3B**) as the cell membranes became more distended during expansion (**Figure 3C**). M-CSF concentration did not have a significant impact on macrophage fusion or size of FBGCs (**Supplementary Figures S2A**, **S3A**). However, whilst interleukins increased the formation of FBGCs containing 4 to 10 nuclei/cell (**Supplementary Figure S3C**), only macrophages differentiated with 100 ng/mL of M-CSF and polarised with IL-4 significantly increased (*p*<0.01) the formation of FBGCs with >10 nuclei/cell (**Supplementary Figure S3E**).

Unlike M-MDMs, there was no significant change in the fusion of GM-MDMs induced by the presence of PET and interleukins (**Figure 3A**). Whilst, neither the GM-CSF concentration nor the combination of interleukins significantly affected FBGC formation by GM-MDMs (**Supplementary Figures S2B**, **S3B, D**), only GM-MDMs differentiated with 2.5 ng/mL of GM-CSF and polarised with IL-13 produced FBGCs containing >10 nuclei/cell (**Supplementary Figure S3F**). No morphological differences were observed between FBGCs formed by either M-MDMs or GM-MDMs in response to IL-4 and/or IL-13 (**Supplementary Figure S2C**).

These results indicate that introducing PET as a foreign biomaterial in combination with interleukins was necessary to induce effective FBGC formation. However, only M-CSF licensed large FBGC formation, which indicates that the macrophage phenotype dictates FBGC formation. The distinct effects on fusion that we observed between macrophages adhered to TCPS and PET also highlights the vital role of the substrate in priming macrophages to fuse following activation of the STAT6 pathway by IL-4 and/or IL-13.

**Figure 4:**
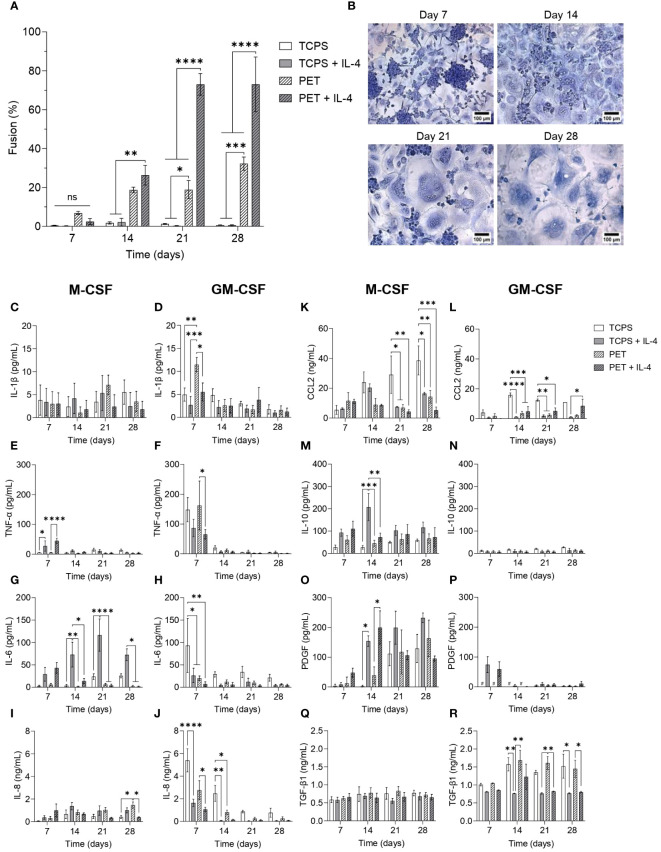
Temporal changes in secretome during FBGC formation **(A)** Macrophage fusion percentage at weekly intervals when macrophages were cultured on TCPS or PET and differentiated with M-CSF (50 ng/mL), with or without IL-4 (10 ng/mL). Data is the mean ± SEM of four biological donors. **(B)** Representative images of fusing macrophages and FBGCs stained with May-Grünwald and Giemsa at weekly intervals when macrophages were cultured on PET with media containing M-CSF and IL-4. Scale bar, 100 μm. **(C–R)** The amount of cytokines and growth factors secreted by M-CSF- and GM-CSF-differentiated macrophages and FBGCs cultured in fusogenic and non-fusogenic conditions was determined by ELISA at weekly intervals. Data is the mean ± SEM of three **(J–L)** to four **(C–I, M–R)** biological donors. Statistical significance was determined at each time point by two-way ANOVA with the Šídák multiple comparison test (*p<0.05, **p<0.01, ***p<0.005, ****p<0.001). ns, not significant.

As the culture surface influenced macrophage morphology and fusion ability, we characterised the initial cell adhesion events occurring when monocytes are seeded on TCPS and PET substrates (**Figures 4E, F**). One hour following cell seeding, we observed a 1.5-fold to 2.3-fold increase in monocytes adhered to PET compared to TCPS in all experimental conditions. In previous studies, the absence of adsorbed serum proteins on biomaterial surfaces (42, 43) and the chelation of calcium by EDTA (44) reduced macrophage fusion. We included similar experimental conditions in our study to determine whether the absence of serum proteins or calcium chelation would reduce initial monocyte adhesion to a fusion-promoting surface. However, we did not observe any statistically significant differences in the extent of monocyte adhesion to TCPS or PET (**Figures 3E, F**).

Given that macrophage morphology and function are known to be influenced by materials’ mechanical and biophysical properties (45), we compared TCPS and PET surface properties to elucidate possible cues that may induce a circular cell shape and enhance monocyte adhesion and subsequent macrophage fusion observed on PET. Both TCPS and PET surfaces exhibited a similar extent of wettability (**Supplementary Figure S4A**). However, TCPS was significantly stiffer (*p*<0.001; **Supplementary Figure S4B**) whilst PET was smoother (*p*<0.05; **Supplementary Figure S4C**) with a different topography (**Supplementary Figure S4D**). The two materials also had different surface chemistries, including the presence of ester bonds in PET and aromatic side chains in TCPS (**Supplementary Figure S4E**), which likely contributed to different biosorption and physical properties that would alter cell response (42).

In summary, monocytes adhered more to PET than TCPS, but the extent of monocyte adhesion to PET reduced when serum proteins were omitted from the culture media. However, characterising the material surface properties revealed no biologically relevant differences between TCPS and PET. This indicates that the contrasting macrophage responses induced by TCPS and PET was likely due to serum protein adsorption as a result of different surface chemistries.

### FBGC formation is associated with a temporal transition from inflammation to wound healing

To elucidate the timescale on which macrophage fusion occurred within our *in vitro* model, cultures were stopped at weekly intervals for up to four weeks. After 7 days of culture, minimal cell fusion occurred (**Figure 4A**), although we observed the appearance of many large cell clusters when macrophages were cultured on PET with IL-4 (**Figure 4B**). After 14 days, relatively similar levels of macrophage fusion were recorded for macrophages cultured on PET with and without IL-4 (**Figure 4A**); however, IL-4 enhanced the formation of larger FBGCs (**Supplementary Figures S5A, B**). By day 21, substantial macrophage fusion (*p*<0.001; **Figure 4A**) and large FBGC formation (**Figures 4B**, **Supplementary Figures S5A**) was observed in response to PET and IL-4. Continuing the FBGC cultures for up to 28 days did not further enhance fusion (**Figure 4A**) nor FBGC size (**Figures 4B**, **Supplementary Figures S5A**).

Once we established that maximal FBGC formation occurred after three weeks of culture, we characterised the secretome of macrophages and FBGCs to identify soluble mediators that may regulate the fusion process. M-MDMs released very low amounts of the pro-inflammatory mediator IL-1β (**Figure 4C**) in all experimental conditions, but had a significantly enhanced production of TNF-α and IL-6 in response to IL-4 (**Figures 4E, G**). GM-MDMs, which were predisposed to a pro-inflammatory phenotype by GM-CSF, also produced these inflammatory mediators, but in contrast to M-MDMs, the addition of IL-4 reduced the secretion of IL-1β, TNF-α and IL-6 by GM-MDMs (**Figures 4D, F, H**). As cell-cell contact is a prerequisite for fusion to occur, we also quantified the production of IL-8 and CCL2 to determine whether macrophage fusion was associated with an elevated expression of pro-inflammatory chemokines involved in cell migration. M-MDMs cultured on PET with IL-4 secreted the greatest amount of IL-8 and CCL2 at day 7 (**Figures 4I, K**), which then reduced over time. However, similar to that observed for the pro-inflammatory cytokines, GM-MDMs produced higher amounts of IL-8 at day 7 than M-MDMs (**Figure 4J**). M-MDMs and GM-MDMs cultured on TCPS also produced greater amounts of CCL2 than macrophages cultured in fusogenic conditions from day 14 onwards (**Figures 4K, L**). These results indicate that whilst M-MDMs cultured on PET with IL-4 exhibited an initial inflammatory response, GM-MDMs also mediated a pro-inflammatory environment at day 7.

We then analysed mediators involved in resolving inflammation and mediating tissue repair to identify any association with FBGC formation. The secretion of IL-10 and PDGF by M-MDMs was significantly enhanced in response to IL-4 at day 14 (**Figures 4M, O**), although IL-10 was only increased when M-MDMs were cultured on TCPS. Conversely, no significant differences in the secretion of TGF-β1 by M-MDMs and FBGCs occurred over time (**Figure 4Q**). In contrast, GM-MDMs produced very low amounts of IL-10 and PDGF regardless of experimental conditions (**Figures 4N, P**) and relatively high amounts of TGF-β1 at day 14 (**Figure 4R**). However, IL-4 significantly reduced the production of TGF-β1 by GM-MDMs (**Figure 4R**).

This analysis of the macrophage and FBGC secretomes during fusion revealed that whilst no cytokine or growth factor was unique to fusing macrophages, FBGC formation is associated with a specific and controlled programme of soluble mediator production that transitioned from pro-inflammation to wound healing. Fusion-competent M-MDMs cultured on PET with IL-4 produced the greatest amount of TNF-α, IL-6, IL-8 and CCL2 compared to M-MDMs cultured in other experimental conditions. Whilst GM-MDMs were also associated with an early production of inflammatory mediators, GM-MDMs failed to adopt a reparative phenotype that was observed by M-MDMs at later time points. Over time, macrophages undergoing fusion facilitated a microenvironment that was conducive to wound healing with a reduced production of pro-inflammatory mediators and substantially increased secretion of PDGF, which coincided with the appearance of large FBGCs.

### Toll-like receptor activation and signalling play a role in the formation of large FBGCs

Under fusogenic conditions, macrophages mounted an early innate immune response featuring a specific programme of pro-inflammatory mediators (**Figures 4C–L**). Macrophages express pattern recognition receptors (PRRs), including Toll-like receptors (TLRs), which are activated upon recognition of pathogens (pathogen-associated molecular patterns (PAMPs)) or endogenous molecules that signal cell damage (damage-associated molecular patterns (DAMPs)) and drive the expression of pro-inflammatory mediators (46). However, little is known about the putative role of TLR activation in FBGC formation. To determine the effect of TLR activation on macrophage fusion, we inhibited and activated TLR signalling by DAMPs and PAMPs.

**Figure 5:**
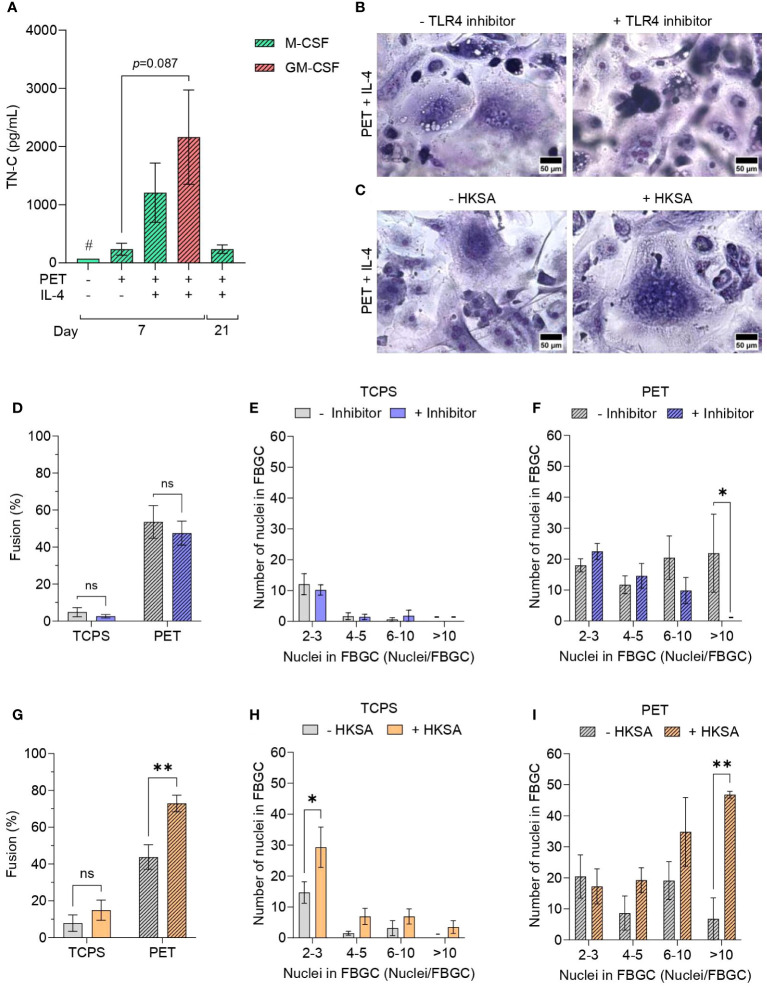
Effect of Toll-like receptor activation and signalling on FBGC formation. **(A)** The amount of tenascin-C (TN-C) secreted by macrophages and FBGCs cultured in fusogenic and non-fusogenic conditions was quantified by ELISA. Macrophages were cultured in M-CSF (50 ng/mL) or GM-CSF (5 ng/mL) with or without IL-4 (10 ng/mL). Data is the mean ± SEM of three biological donors, except for the TN-C control condition denoted with (#) which represents one biological donor. No statistical significance was determined by two-way ANOVA with the Tukey multiple comparison test. Representative images of May-Grünwald/Giemsa-stained M-MDMs and FBGCs cultured in fusogenic conditions in the presence and absence of **(B)** TLR4 inhibitor TAK-242 and **(C)** TLR activator HKSA. Scale bar, 50 μm. **(D)** Percentage of macrophage fusion on TCPS and PET in response to TLR4 inhibition. FBGC size in response to TLR4 inhibition according to number of nuclei per FBGC when macrophages cultured on **(E)** TCPS or **(F)** PET. Data is the mean ± SEM of five biological donors. **(G)** Percentage of macrophage fusion on TCPS and PET in response to TLR activation by HKSA. FBGC size following TLR activation by HKSA according to the number of nuclei/FBGC when macrophages were cultured on **(H)** TCPS or **(I)** PET. **(B–I)** Macrophages and FBGCs were cultured in M-CSF (50 ng/mL) and IL-4 (10 ng/mL). Data is the mean ± SEM of three (PET) and four (TCPS) biological donors. Statistical significance was determined by two-way ANOVA with the Šídák multiple comparison test (*p<0.05, **p<0.01). ns, not significant.

First, we determined the presence of a DAMP in the microenvironment surrounding macrophages. DAMPs including HMGB1, HSPs, S100 proteins and annexins, which are intracellular molecules normally inaccessible to the immune system that are released following cell necrosis or cell activation upon injury, were reported previously to adsorb to biomaterial surfaces and activate macrophages (47, 48). However, other DAMPs, including ECM molecules that are specifically upregulated in response to tissue injury, have not yet been studied in connection with FBGC formation. Here, we quantified the production of the pro-inflammatory ECM glycoprotein tenascin-C (TN-C), which is transiently expressed upon tissue injury and induces cytokine synthesis via TLR4 activation in myeloid cells (15). As expected, pro-inflammatory GM-MDMs produced the greatest amount of TN-C; however, culturing macrophages in fusion-permissive conditions did not induce any statistically significant differences in TN-C secretion (**Figure 5A**).

Next, we investigated whether TLR4 blockade would impair macrophage fusion. Although TAK-242, an inhibitor of TLR4 signal transduction, did not affect total macrophage fusion (**Figure 5D**) or the size of FBGCs formed on TCPS (**Figure 5E**), macrophages on PET were unable to form large FBGCs (>10 nuclei/cell) in the presence of TAK-242 (*p*<0.05; **Figures 5B, F**).

We subsequently investigated the impact of PAMP-mediated TLR signalling on macrophage fusion by activating macrophage TLRs with heat-inactivated *Staphylococcus aureus* (HKSA) (49). The addition of HKSA to macrophage cultures only increased the percentage of macrophage fusion when M-MDMs were cultured in fusion-permission conditions (*p*<0.01; **Figure 5G**). Macrophages cultured on TCPS had an increased formation of small (2–3 nuclei/cell) multinucleated cells (*p*<0.05; **Figure 5H**) in the presence of HKSA. Conversely, culturing macrophages on PET with HKSA significantly increased the formation of large FBGCs (*p*<0.01; **Figures 5C, I**).

In brief, FBGC formation was in part mediated by TLR activation by DAMP and PAMP signals, with TLR inhibition and activation resulting in reduced and enhanced formation of large FBGCs, respectively.

### Fibroblast co-culture enhances FBGC formation

**Figure 6:**
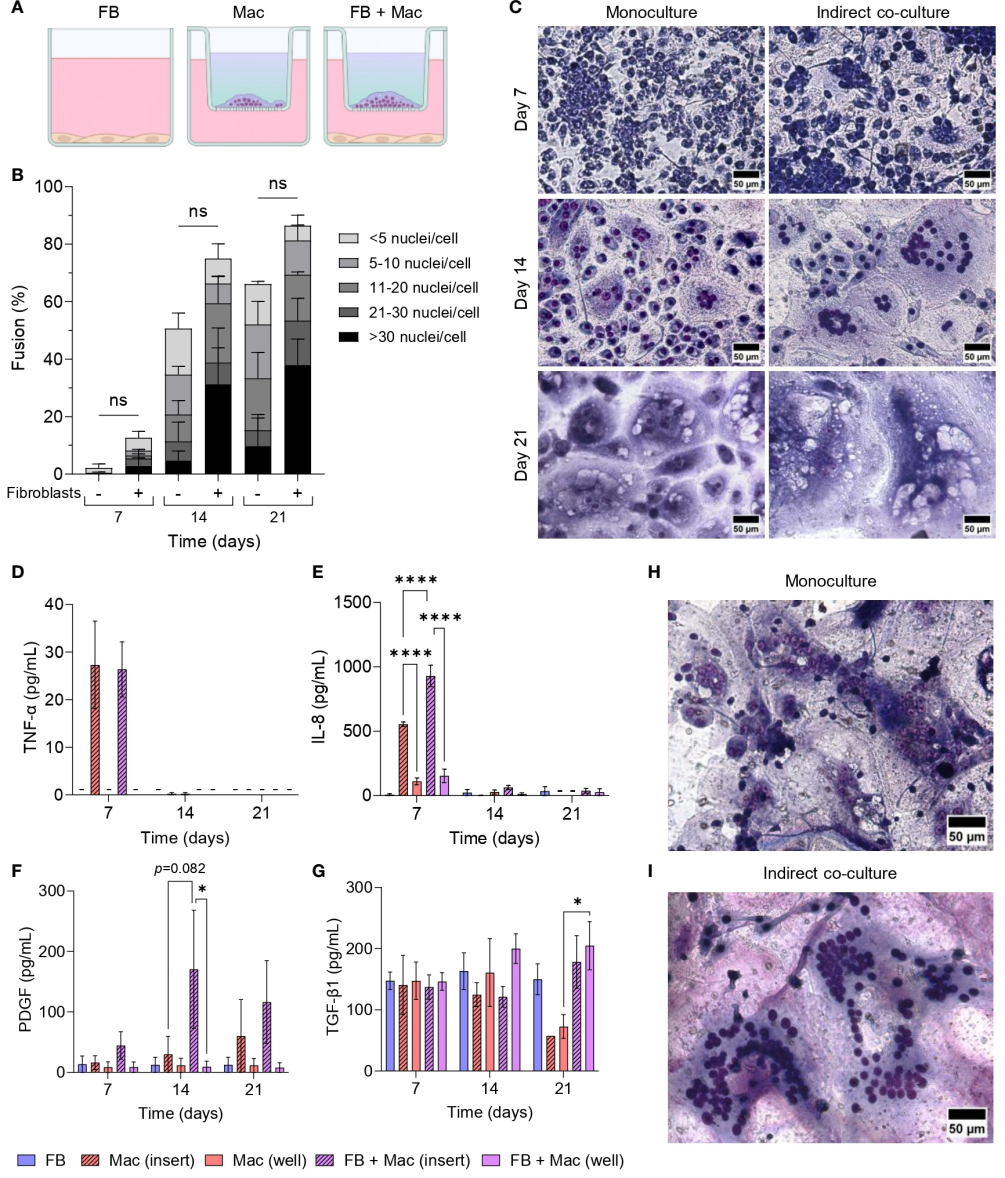
Effect of fibroblasts on FBGC formation in macrophage-fibroblast indirect co-cultures. **(A)** Schematic of indirect co-culture set-up. Fibroblasts (FB) were seeded into wells and cultured in media without cytokine supplementation. Monocytes were seeded into PET inserts and differentiated to macrophages (Mac) with M-CSF (50 ng/mL). IL-4 (10 ng/mL) was added to monocytes after 72 hours to induce FBGC formation. The combination of M-CSF and IL-4 in the medium is depicted as a blue-green gradient within the insert. **(B)** Percentage of macrophage fusion at weekly intervals with stacked bars showing the percentage of nuclei within FBGC containing a total of<5, 6 to 10, 11 to 20, 21 to 30 and/or >30 nuclei/cell. **(C)** Representative images of May-Grünwald/Giemsa-stained macrophages and FBGCs in monocultures and indirectly co-cultured with fibroblasts at weekly intervals. Scale bar, 50 μm. **(D–G)** Secretion of cytokines and growth factors determined by ELISA from macrophages and FBGCs in monoculture and indirect co-culture with fibroblasts. Supernatants were obtained from both inserts and wells. Data is the mean ± SEM of three **(E)**, four **(D, F)** and five **(G)** biological donors. Statistical significance was determined by two-way ANOVA with the Šídák multiple comparison test (*p<0.05, ****p<0.001). Representative images of the largest syncytium formed in **(H)** macrophage monocultures and **(I)** macrophage-fibroblast indirect co-cultures. Macrophages/FBGCs were stained with May-Grünwald/Giemsa and imaged after 14 days. The largest cell mass contained 86 and 177 nuclei in monoculture and co-culture, respectively. Scale bar, 50 μm. ns, not significant.

As both macrophages and fibroblasts are critical cells involved in the progression of the FBR, we established a series of monocyte/macrophage-fibroblast co-cultures (**Figure 1**) to determine whether fibroblasts may contribute to FBGC formation. To assess the impact of macrophage-fibroblast crosstalk via paracrine and autocrine signals on FBGC formation, an indirect co-culture system was developed by culturing fibroblasts in TCPS wells whilst monocytes were seeded on PET inserts and differentiated into macrophages with M-CSF (**Figure 6A**). Co-culturing macrophages with fibroblasts did not significantly affect FBGC formation (**Figure 6B**, **Supplementary Figures S6A–D**). However, at day 14 we observed a trend towards enhanced macrophage fusion and large FBGC formation in co-cultures (*p*<0.1, **Supplementary Figures S6A, B**; **Figures 6B, C**). Expansive syncytia were also observed at day 14 wherein co-cultures contained up to 177 nuclei in one cell mass (**Figure 6I**), whereas the largest FBGC that was detected in monocultures contained 86 nuclei (**Figure 6H**).

Supernatants from both inserts and wells were sampled to determine whether there was any change in the production of soluble mediators due to co-culturing macrophages and FBGCs with fibroblasts. TNF-α and IL-8 were primarily detected in insert supernatants (**Figures 6D, E**), indicating that macrophages were the primary producers of these mediators. Interestingly, macrophages within the co-culture system had an enhanced production of IL-8 at day 7 (*p*<0.001, **Figure 6E**) and PDGF at day 14 (*p*=0.082, **Figure 6F**) compared to macrophages in monocultures. In contrast, TGF-β1 remained largely unchanged until day 21, when its levels raised in co-cultures compared to macrophage monocultures (**Figure 6G**).

**Figure 7:**
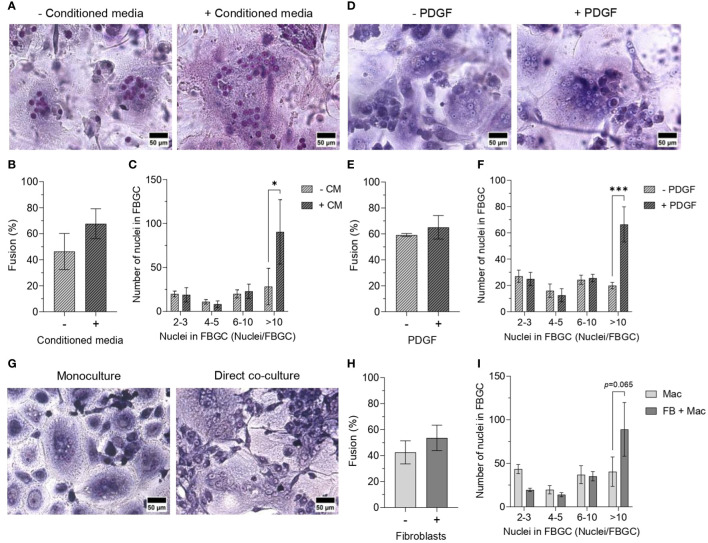
Effect of fibroblast-conditioned medium and fibroblast-macrophage contact on FBGC formation. Representative images of macrophages and FBGCs stained with May-Grünwald and Giemsa after 21 days of culture on PET in M-CSF (50 ng/mL) and IL-4 (10 ng/mL) without or with **(A)** fibroblast-conditioned media (CM; diluted 1:1 with fresh media) and **(D)** PDGF (100 pg/mL from day 7, 200 pg/mL from day 10, 400 pg/mL from day 17). Scale bar, 50 μm. Percentage of macrophage fusion after 21 days of culture on PET in M-CSF and IL-4 with the addition of **(B)** conditioned media or **(E)** PDGF. FBGC size according to number of nuclei per FBGC when macrophages were cultured for 21 days on PET with M-CSF and IL-4 with the addition of **(C)** conditioned media and **(F)** PDGF. **(G)** Representative images of macrophages and FBGCs directly co-cultured with fibroblasts in media containing M-CSF (50 ng/mL) and IL-4 (10 ng/mL) for 14 days. Macrophages, FBGCs and fibroblasts were stained with May-Grünwald and Giemsa. Scale bar, 50 μm. **(H)** Percentage of macrophage fusion and **(I)** FBGC size according to number of nuclei/FBGC when macrophages were directly co-cultured with fibroblasts in medium containing M-CSF and IL-4 for 14 days. Data is the mean ± SEM of three **(E, F)** to four **(B, C, H, I)** biological donors. No statistical significance for **(B, E, H)** was determined by unpaired t-tests. Statistical significance for **(C, F, I)** was determined by two-way ANOVA with the Šídák multiple comparison test (*p<0.05, ***p<0.005).

We next cultured macrophages in conditioned medium from fibroblast monocultures to determine whether macrophage fusion could be enhanced by fibroblast-secreted mediators. The addition of fibroblast-conditioned medium (CM) did not significantly affect macrophage fusion (**Figure 7B**); however, fibroblast-CM did increase the formation of large FBGCs containing >10 nuclei/cell (*p*<0.05; **Figures 7A, C**). Fibroblast-CM was also added to macrophages cultured on TCPS and PET without IL-4 to determine whether macrophage fusion could be initiated in the absence of a foreign substrate and IL-4. No effect on macrophage fusion (**Supplementary Figure S7B**) or FBGC size (**Supplementary Figures S7C, D**) was observed by the addition of fibroblast-CM, which indicated that fibroblast soluble mediators only contribute to enhancing FBGC formation in fusion-permissive conditions. Fibroblast-CM also caused macrophages on TCPS to adopt an elongated morphology, whilst macrophages on PET exhibited no morphological changes (**Supplementary Figure S7A**).

As enhanced PDGF secretion coincided with the time scale observed for large FBGC formation in mono- (**Figure 4O**) and co-cultures (**Figure 6F**), fusing macrophages were supplemented with recombinant PDGF to determine whether PDGF facilitates macrophage fusion. Similar to culturing macrophages in fibroblast-CM, only the formation of large FBGCs (>10 nuclei/cell) was significantly enhanced (*p*<0.005; **Figures 7D, F**) despite there being a minimal difference in the total percentage of macrophage fusion (**Figure 7E**).

Finally, to elucidate the contribution of direct cell-cell interactions between macrophages and fibroblasts towards macrophage fusion, macrophages were seeded on a fibroblast monolayer on TCPS and cultured in a cocktail of M-CSF and IL-4 to induce fusion (**Figure 7G**). Similarly, the total percentage of macrophage fusion was relatively unaffected (**Figure 7H**), whilst there was a trend towards increased large FBGC formation (>10 nuclei/cell) when macrophages were directly co-cultured with fibroblasts (p=0.065, **Figure 7I**).

In summary, exposing fusing macrophages to soluble mediators produced by fibroblasts increased the formation of large FBGCs. IL-8 and PDGF secretion was enhanced when macrophages and FBGCs were indirectly co-cultured with fibroblasts. Supporting our earlier observations of FBGC formation coinciding with increased PDGF secretion, exogenous PDGF also enhanced large FBGC formation.

## Discussion

**Figure 8:**
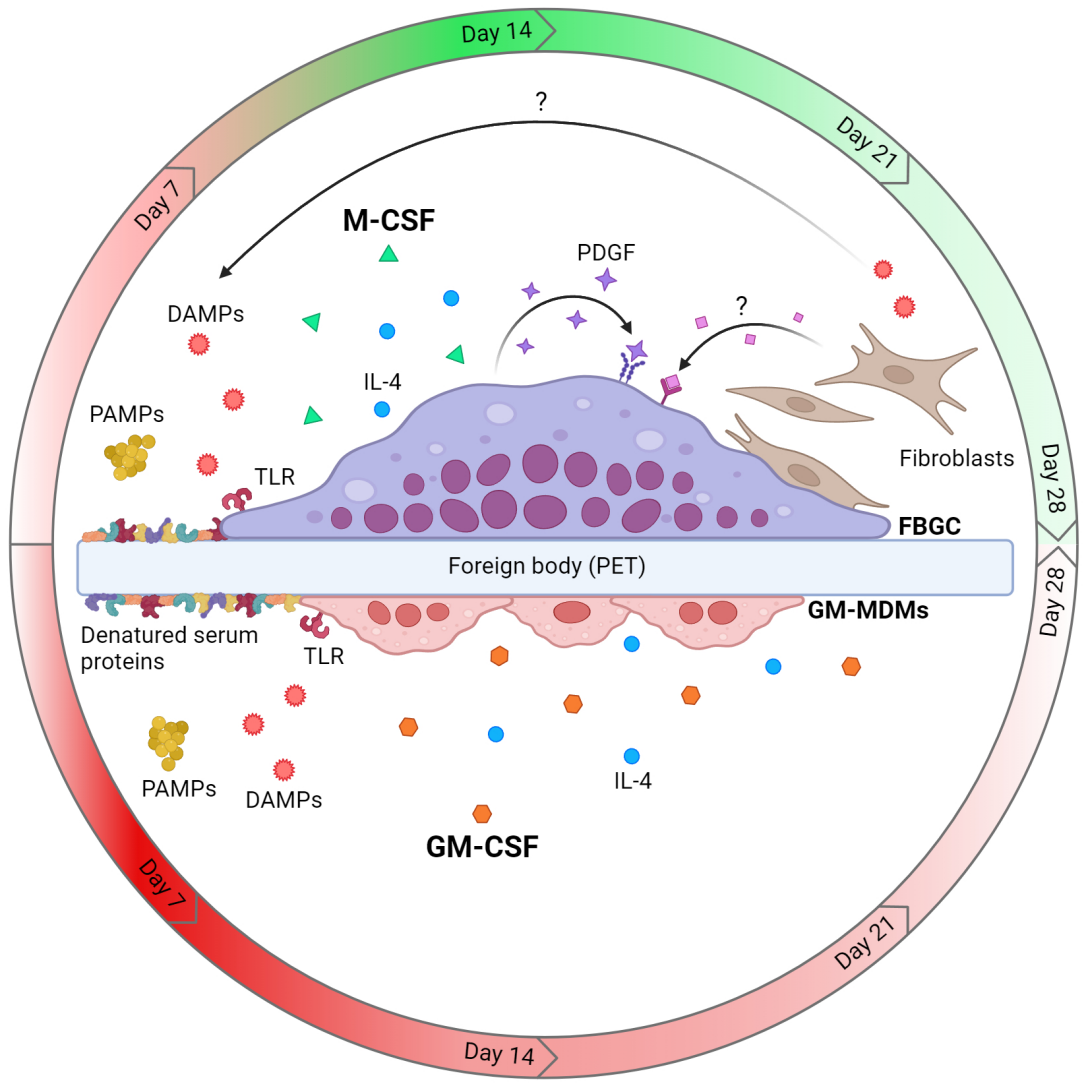
Model of cellular and microenvironmental cues that dictate macrophage fusion and FBGC formation. Macrophages differentiated with M-CSF are fusion competent and can form large FBGCs in the presence of IL-4 and a foreign body (e.g. polyethylene terephthalate (PET)), whereas GM-CSF-mediated differentiation does not licence macrophages to form FBGCs. Fusing M-MDMs and FBGCs are associated with an early pro-inflammatory response which transitions to tissue repair by day 14 (red and green gradients, respectively), whilst GM-MDMs maintain an inflammatory phenotype (red gradient). Activation of TLRs by PAMPs (e.g. bacterial ligands) or DAMPs (e.g. TN-C and denatured serum proteins adsorbed to biomaterial) allows the formation of large FBGCs. Finally, the autocrine growth factor PDGF and soluble paracrine factors secreted by fibroblasts also enhance the formation of large FBGCs. Created with BioRender.com.

Macrophage multinucleation is a complex and dynamic process in which manifold microenvironmental factors dictate FBGC formation in the context of the FBR. However, the effect of microenvironmental cues at the cellular and tissue level on FBGC formation is poorly understood. This study investigated key factors implicated in programming macrophage activities during the FBR and their effect on FBGC formation *in vitro*. We showed that monocyte differentiation influenced the ability of macrophages to adopt a fusion competent state and FBGC formation was associated with specific and temporal microenvironmental cues. Large FBGCs were distinctly associated with an initial inflammatory response likely involving TLR activation that transitioned into wound healing activities mediated by autocrine and paracrine signalling of growth factors. We also identified a notable contribution of the fibroblast secretome and fibroblast-macrophage cell contact in driving FBGC formation. The microenvironmental cues that promoted FBGC formation are multifactorial and can occur simultaneously to drive the FBR (**Figure 8**).

Within 24 hours of biomaterial implantation, circulating monocytes are recruited to the site of tissue injury (50). Monocyte differentiation into functional mature macrophages has been overlooked in the investigation of FBGC formation. Whilst studies have employed either M-CSF (2, 36) or GM-CSF (24, 25, 35) to induce monocyte differentiation prior to FBGC formation, we identified that M-CSF and GM-CSF have distinct effects on programming macrophages to adopt a fusion-competent state.

M-CSF induced very low levels of fusion that could not generate true FBGCs. The addition of IL-4 and IL-13, which induce macrophage fusion via the STAT6 pathway (20), enhanced macrophage fusion and formation of FBGCs. Notably, FBGC development positively correlated with M-CSF concentration, suggesting that macrophage maturation influences their fusion competence. Accordingly, β1 integrins, which are only expressed by mature macrophages and FBGCs, have been shown to facilitate macrophage fusion (27). Furthermore, M-MDMs formed larger FBGCs in response to IL-4 alone, regardless of substrate, which may be due to IL-4 activating the STAT6 pathway through IL4Rα more effectively than IL-13 via IL13Rα1 (39).

In stark contrast, increasing concentrations of GM-CSF reduced FBGC formation by GM-MDMs following IL-4 polarisation. Numerous studies have used GM-CSF to differentiate MDMs prior to FBGC formation despite GM-CSF in combination with IL-4 inducing dendritic cell formation (51). Accordingly, our GM-MDM cultures polarised with IL-4 displayed morphologies characteristic of dendritic cells with cytoplasmic projections, reduced cell density and lack of long-term (>14 days) survival (52). As dendritic cells do not participate in cell-cell fusion, lower GM-CSF concentrations may impair dendritic cell formation following IL-4 polarisation, favouring monocyte differentiation into macrophages, as we observed. In further contrast to M-CSF, FBGC formation by GM-MDMs was more effective in response to IL-13. The combination of GM-CSF and IL-13 may have skewed the differentiation of monocytes into macrophages as IL-13 induces dendritic cell formation less efficiently than IL-4 (53).

In our study, TCPS did not support effective FBGC formation by macrophages and we sought to incorporate a model biomaterial that invokes a strong FBR. Whilst PET is one of the most commonly used polymers in biomedical applications (54), PET implants are reported to induce a local foreign body reaction that can eventually result in fibrosis (55, 56). Consequently, we utilised PET as a model biomaterial to study FBGC formation *in vitro*. The PET biomaterial further differentiated the ability of M-CSF and GM-CSF to facilitate FBGC formation in the presence of polarising interleukins. Only M-CSF-differentiated macrophages on PET underwent high levels of fusion and formed large FBGCs. PET also reduced the appearance of dendritic cell morphologies in GM-MDM cultures, possibly inducing a monocyte activation state that favours macrophage over dendritic cell differentiation. One reason for the discrepancy between M-CSF and GM-CSF may be the differential expression of fusion mediators such as dendritic cell-specific transmembrane protein (DC-STAMP) and the M-CSF receptor (CSF-1R). GM-CSF was reported to abolish DC-STAMP expression (26) and cleaved the CSF-1R on macrophages (57), preventing macrophage fusion. Together, these data reveal that GM-CSF induces a macrophage program that impedes FBGC formation, whereas M-CSF enables macrophages to respond effectively to fusogenic stimuli forming large FBGCs as *in vivo* (40, 58).

The surface properties of TCPS and PET substrates, including surface wettability, roughness, and substrate stiffness, were not driving the substantial differences observed in the macrophage response. It is possible that the chemical composition of TCPS and PET distinctively affects serum protein adsorption that influences macrophage fusion. The adsorption of serum proteins to biomaterials is known to influence monocyte adhesion, macrophage activation and FBGC formation (29, 43) as denatured adsorbed proteins act as danger signals to inflammatory cells (48). In our study, we did not observe any statistically significant differences in the extent of monocyte adhesion to biomaterial surfaces. However, the composition and/or denaturation of serum proteins that adsorb to PET may induce a stronger inflammatory response in monocytes and macrophages. Variations in the adsorbed protein composition and/or denaturation between TCPS and PET could be due to differences in chemical composition of the substrates. The high prevalence and accessibility of ester groups in PET may enable it to engage in stronger interactions with adsorbed serum proteins (e.g. via hydrogen bonds), increasing the likelihood of protein denaturation. In contrast, TCPS possesses a denser arrangement of aromatic groups that may restrict access to its polar groups, possibly reducing its ability to interact with adsorbed proteins.

The theory that PET induces a greater inflammatory response, likely via denatured adsorbed proteins, in monocytes and macrophages is further supported by the cell morphologies that we observed. M-MDMs on TCPS were relatively small with a round or elongated shape, which indicates a neutral or alternatively-activated macrophage phenotype (37). In contrast, M-MDMs on PET adopted large and round, ‘pancake-like’ morphologies characteristic of pro-inflammatory macrophages (37), which were also observed in GM-CSF-differentiated macrophages regardless of substrate. Moreover, we did not observe dendritic cell morphologies in response to GM-CSF and IL-4 when monocytes were cultured on PET instead of TCPS. Preferential macrophage differentiation when monocytes adhered to PET might be the result of biomaterial-induced activation of the TLR pathway. Indeed, when Xie et al. activated day 0 monocytes with lipopolysaccharide followed by GM-CSF and IL-4, dendritic cell differentiation was prevented (59). Our investigation of the role of TLR signalling in FBGC formation revealed that TLR activation by PAMP ligands increased macrophage fusion and formation of large FBGCs. Correspondingly, inhibition of TLR4 signalling significantly reduced the formation of large FBGCs. Whilst a deeper investigation of the underlying molecular mechanism is required, our data suggest that TLR signalling is a prerequisite to inducing FBGC formation on permissive substrates such as PET and it can be activated by distinct signals, including cell-derived DAMPs, biomaterial-associated DAMPs (i.e. denatured, adsorbed serum proteins) and microbial ligands.

Our analysis of the temporal changes in the macrophage secretome during fusion further elucidated the microenvironmental cues implicated in FBGC formation. In support of our data suggesting that PET induces an inflammatory response in macrophages, GM-MDMs cultured on PET produced the greatest amounts of IL-1β and TNF-α by day 7. In contrast, M-MDMs cultured on PET produced less TNF-α, IL-6 and IL-8, which indicates that macrophages did not adopt a highly pro-inflammatory phenotype in response to PET alone. However, the addition of IL-4 to M-MDMs on PET increased the early production of TNF-α, IL-6 and IL-8, all of which are implicated in acute inflammatory activities. Whilst IL-4 polarisation is typically associated with anti-inflammatory activities, IL-4 is also reported to potentiate the macrophage response to inflammatory or pathogenic stimuli (60). In line with this, we anticipate that TLR activation primes macrophages towards an inflammatory phenotype that is enhanced following IL-4 polarisation. IL-8 mediates cell recruitment and migration during tissue injury and the enhanced production in fusogenic conditions at day 7 coincided with substantial cell aggregation that was only observed in macrophages primed to fuse. As such, the release of this chemokine may facilitate cell-cell contact which is fundamental for fusion to occur. Cell aggregation also facilitates the expression and distribution of β integrins by macrophages prior to fusion. For instance, cell aggregation was reported to change the localisation of β integrins from a diffuse distribution in non-fusing macrophages to clustering at the cell periphery in fusing macrophages and FBGCs (27, 61). In our study, it is possible that chemokine-driven cell aggregation initiated a change in integrin distribution that is reported to facilitate cell fusion and formation of large FBGCs. Moreover, as the peripheral distribution of integrins was also associated with increased cell spreading (61), this coincides with the greater cytoplasmic spreading and reduced cell circularity that we observed in large FBGCs formed in highly fusogenic conditions.

The inflammatory microenvironment generated by macrophages under fusogenic conditions in week one shifted towards a wound healing microenvironment. Unlike GM-MDMs, M-MDMs secreted IL-10 at all-time points, and at the onset of FBGC formation (day 14) they produced the highest amount of PDGF. This suggests that GM-MDMs failed to produce large FBGCs as GM-CSF sustained a pro-inflammatory microenvironment that prevented the phenotypic transition of macrophages implicated in FBGC formation (31). As FBGC formation became prominent (day 21), PDGF secretion by fusing M-MDMs started to decline, which may reflect FBGCs adopting a more quiescent role as the FBR attempts to isolate the foreign material and facilitate tissue homeostasis (50). Surprisingly, GM-MDMs secreted greater amounts of TGF-β1 compared to M-MDMs. At first, this was unexpected, due to TGF-β1 being a master regulator of wound healing (62), however, TGF-β1 exerts pleiotropic effects depending on the cell microenvironment (63). Whilst no specific cytokine or growth factor was identified to drive macrophage fusion, a specific and temporal programme of soluble mediator production was associated with FBGC formation *in vitro.* This observation aligns with the shift from a pro-inflammatory to alternatively-activated macrophage phenotype that has been reported on day 14 of the FBR *in vivo* (16).

Not only macrophages, but also fibroblasts play critical roles in wound healing with macrophages regulating inflammation and mediating the fibrotic encapsulation of biomaterials (58) and fibroblasts synthesising and remodelling new ECM following tissue injury (14). How fibroblasts influence FBGC formation is unclear and co-culturing macrophages with fibroblasts has yielded conflicting results (64–66). Using an indirect co-culture model that enabled the exchange of soluble mediators between fusing macrophages and fibroblasts, we observed that at day 14 macrophages in co-culture displayed a trend towards increased FBGC formation and were capable of forming extremely large syncytia (>100 nuclei/cell). Co-cultured macrophages also had an increased production of IL-8 and PDGF. IL-8 may facilitate cell-cell contact prior to fusion, as discussed above, while PDGF may facilitate the establishment of a microenvironment conducive to FBGC formation. We also observed increased TGF-β1 production at day 21 in line with the study by Pierce et al. where treatment of rat fibroblasts with PDGF increased TGF-β mRNA and protein production (67). Exogenously added PDGF substantially increased the formation of large FBGCs (>10 nuclei/cell), indicating that PDGF secreted by macrophages may act in an autocrine manner to drive the formation of FBGCs. Similarly, large FBGC formation was also enhanced when macrophages were cultured in fibroblast-conditioned medium. However, fibroblasts in monoculture produced low amounts of PDGF, thus other soluble factors might be implicated in driving large FBGC formation. Fibroblasts are mechanosensitive and can become activated in response to stiff tissue-culture plasticware (68) and produce inflammatory cytokines (69) and DAMPs such as TN-C (70). Such inflammatory stimuli may prime macrophages to detect a foreign substrate, enhancing FBGC formation. This is supported by recent findings in which fibroblasts produced DAMPs that adsorbed to biomaterials in competition with plasma proteins and induced an inflammatory response in murine macrophages via TLR2 activation (71).

There remain a number of outstanding questions. For instance, whilst we identified that M-CSF and GM-CSF induced different macrophage responses that permitted and prevented FBGC formation, respectively, it will be important to examine whether M-CSF and GM-CSF induce different macrophage programs that facilitate or abrogate FBGC formation. Another key question is what specific cues are provided by PET that trigger macrophage fusion and how. The answer may involve the extent and composition of protein deposition to the biomaterial and macrophage polarisation, which have been found to be closely linked (23). To this end, it will be important to determine the identity and origins of DAMPs that drive activation of TLRs and examine which specific TLR(s) and downstream signalling pathways are involved in macrophage fusion and large FBGC formation. Moreover, it is unknown whether a temporal activation of TLRs permits FBGC formation or if continuous stimulation is required to signal the persistence of a foreign body and progress the immune reaction towards the chronic FBR stage. Finally, this is the first report that demonstrates fibroblasts can directly influence the development of FBGCs in the FBR. Here, we focused on distinct types of fibroblast-macrophage interactions and their effect on FBGC formation. Future work should identify specific mediators synthesised by fibroblasts, including DAMPs that may enhance the initial inflammatory response of macrophages to foreign bodies (70, 71).

In conclusion, our study highlights key microenvironmental cues ranging from monocyte differentiation to a temporal transition of macrophage phenotype that are implicated in the initiation and progression of the FBR. A specific combination of stimuli programs macrophages to adopt a fusion-competent state and indicates that FBGC formation is a tightly controlled phenomenon. Whilst the FBR is centred upon the presence of a foreign biomaterial, it is the subsequent biological processes that are responsible for permitting and driving FBGC formation in the FBR. This enhanced biological understanding of how microenvironmental cues at the cellular and tissue level influence FBGC formation may provide greater insight into strategies for modulating the FBR. For instance, characterising the biomaterial adsorption of DAMPs, targeting M-CSF signaling, or modulating fibroblast activities may be explored to manipulate the FBR advantageously in the design of therapeutic biomaterials.

## Data availability statement

The original contributions presented in the study are included in the article/**Supplementary Material**. Further inquiries can be directed to the corresponding author/s.

## Ethics statement

Ethics approval or specific consent procedures were not required for this study as it used anonymised human tissue samples (i.e., peripheral blood) obtained from the NHS Blood and Transplant. 

## Author contributions

CS: Conceptualization, Data curation, Formal analysis, Investigation, Methodology, Project administration, Validation, Visualization, Writing – original draft, Writing – review & editing. AH: Formal analysis, Methodology, Supervision, Writing – review & editing. MZ: Formal analysis, Methodology, Supervision, Writing – review & editing. MM: Conceptualization, Funding acquisition, Supervision, Writing – review & editing. AP: Conceptualization, Data curation, Formal analysis, Methodology, Project administration, Resources, Supervision, Validation, Visualization, Writing – original draft, Writing – review & editing.

## References

[1] Galli SJ Borregaard N Wynn TA. Phenotypic and functional plasticity of cells of innate immunity: macrophages, mast cells and neutrophils. Nat Immunol. (2011) 12:1035–44. doi: 10.1038/ni.2109 PMC3412172 22012443

[2] Ahmadzadeh K Pereira M Vanoppen M Bernaerts E Ko J-H Mitera T. Multinucleation resets human macrophages for specialized functions at the expense of their identity. EMBO Rep. (2023) 24:e57070. doi: 10.15252/embr.202357070 37016941 PMC10074087

[3] Takito J Nakamura M. Heterogeneity and actin cytoskeleton in osteoclast and macrophage multinucleation. Int J Mol Sci. (2020) 21:6629–59. doi: 10.3390/ijms21186629 PMC7554939 32927783

[4] Ahmadzadeh K Vanoppen M Rose CD Matthys P Wouters CH. Multinucleated giant cells: current insights in phenotype, biological activities, and mechanism of formation. Front Cell Dev Biol. (2022) 10:873226. doi: 10.3389/fcell.2022.873226 35478968 PMC9035892

[5] Pereira M Petretto E Gordon S Bassett JHD Williams GR Behmoaras J. Common signalling pathways in macrophage and osteoclast multinucleation. J Cell Sci. (2018) 131:jcs216267. doi: 10.1242/jcs.216267 29871956

[6] Klopfleisch R Jung F. The pathology of the foreign body reaction against biomaterials. J BioMed Mater Res A. (2017) 105:927–40. doi: 10.1002/jbm.a.35958 27813288

[7] Klopfleisch R. Macrophage reaction against biomaterials in the mouse model - Phenotypes, functions and markers. Acta Biomater. (2016) 43:3–13. doi: 10.1016/j.actbio.2016.07.003 27395828

[8] Lemaire I Falzoni S Adinolfi E. Purinergic signaling in giant cell formation. Front Biosci. (2012) 4:41–55. doi: 10.2741/e359 22201854

[9] Yadav TC Bachhuka A. Tuning foreign body response with tailor-engineered nanoscale surface modifications: fundamentals to clinical applications. J Mater Chem B Mater Biol Med. (2023) 11:7834–54. doi: 10.1039/D3TB01040F 37528807

[10] Morris AH Mahal RS Udell J Wu M Kyriakides TR. Multicompartment drug release system for dynamic modulation of tissue responses. Adv Healthc Mater. (2017) 6:1700370. doi: 10.1002/adhm.201700370 28636088

[11] Anderson JM Rodriguez A Chang DT. Foreign body reaction to biomaterials. Semin Immunol. (2008) 20:86–100. doi: 10.1016/j.smim.2007.11.004 18162407 PMC2327202

[12] Sheikh Z Brooks PJ Barzilay O Fine N Glogauer M. Macrophages, foreign body giant cells and their response to implantable biomaterials. Materials. (2015) 8:5671–701. doi: 10.3390/ma8095269 PMC5512621 28793529

[13] Zhao Q Topham N Anderson JM Hiltner A Lodoen G Payet CR. Foreign-body giant cells and polyurethane biostability: in *vivo* correlation of cell adhesion and surface cracking. J BioMed Mater Res. (1991) 25:177–83. doi: 10.1002/jbm.820250205 2055915

[14] Witherel CE Abebayehu D Barker TH Spiller KL. Macrophage and fibroblast interactions in biomaterial-mediated fibrosis. Adv Healthc Mater. (2019) 8:e1801451. doi: 10.1002/adhm.201801451 30658015 PMC6415913

[15] Piccinini AM Zuliani-Alvarez L Lim JMP Midwood KS. Distinct microenvironmental cues stimulate divergent TLR4-mediated signaling pathways in macrophages. Sci Signal. (2016) 9:ra86. doi: 10.1126/scisignal.aaf3596 27577261 PMC5338747

[16] Saleh LS Amer LD Thompson BJ Danhorn T Knapp JR Gibbings SL. Mapping Macrophage Polarization and Origin during the Progression of the Foreign Body Response to a Poly(ethylene glycol) Hydrogel Implant. Adv Healthc Mater. (2022) 11:e2102209. doi: 10.1002/adhm.202102209 34967497 PMC9081184

[17] Jones JA McNally AK Chang DT Qin LA Meyerson H Colton E. Matrix metalloproteinases and their inhibitors in the foreign body reaction on biomaterials. J BioMed Mater Res A. (2008) 84:158–66. doi: 10.1002/jbm.a.31220 17607751

[18] Jones K. Chapter 9 - Fibrotic Response to Biomaterials and all Associated Sequence of Fibrosis. In: Badylak SF, editor. Host Response to Biomaterials. London, UK: Oxford: Academic Press (2015). p. 189–237.

[19] Jansen LE Amer LD Chen EY-T Nguyen TV Saleh LS Emrick T. Zwitterionic PEG-PC hydrogels modulate the foreign body response in a modulus-dependent manner. Biomacromolecules. (2018) 19:2880–8. doi: 10.1021/acs.biomac.8b00444 PMC6190668 29698603

[20] Helming L Gordon S. Molecular mediators of macrophage fusion. Trends Cell Biol. (2009) 19:514–22. doi: 10.1016/j.tcb.2009.07.005 19733078

[21] Trout KL Holian A. Factors influencing multinucleated giant cell formation in *vitro* . Immunobiology. (2019) 224:834–42. doi: 10.1016/j.imbio.2019.08.002 PMC6874761 31439452

[22] Zhu F Wang S Zhu X Pang C Cui P Yang F. Potential effects of biomaterials on macrophage function and their signalling pathways. Biomater Sci. (2023) 11:6977–7002. doi: 10.1039/D3BM01213A 37695360

[23] Rostam HM Fisher LE Hook AL Burroughs L Luckett JC Figueredo GP. Immune-instructive polymers control macrophage phenotype and modulate the foreign body response in vivo. Matter. (2020) 2:1564–81. doi: 10.1016/j.matt.2020.03.018

[24] McNally AK Anderson JM. Interleukin-4 induces foreign body giant cells from human monocytes/macrophages. Differential lymphokine regulation of macrophage fusion leads to morphological variants of multinucleated giant cells. Am J Pathol. (1995) 147:1487–99. PMC1869534 7485411

[25] DeFife KM Jenney CR McNally AK Colton E Anderson JM. Interleukin-13 induces human monocyte/macrophage fusion and macrophage mannose receptor expression. J Immunol. (1997) 158:3385–90. doi: 10.4049/jimmunol.158.7.3385 9120298

[26] Yagi M Miyamoto T Sawatani Y Iwamoto K Hosogane N Fujita N. DC-STAMP is essential for cell–cell fusion in osteoclasts and foreign body giant cells. J Exp Med. (2005) 202:345–51. doi: 10.1084/jem.20050645 PMC2213087 16061724

[27] McNally AK Anderson JM. Beta1 and beta2 integrins mediate adhesion during macrophage fusion and multinucleated foreign body giant cell formation. Am J Pathol. (2002) 160:621–30. doi: 10.1016/S0002-9440(10)64882-1 PMC1850662 11839583

[28] Podolnikova NP Kushchayeva YS Wu Y Faust J Ugarova TP. The role of integrins αMβ2 (Mac-1, CD11b/CD18) and αDβ2 (CD11d/CD18) in macrophage fusion. Am J Pathol. (2016) 186:2105–16. doi: 10.1016/j.ajpath.2016.04.001 PMC4973655 27315778

[29] Shen M Garcia I Maier RV Horbett TA. Effects of adsorbed proteins and surface chemistry on foreign body giant cell formation, tumor necrosis factor alpha release and procoagulant activity of monocytes. J BioMed Mater Res A. (2004) 70:533–41. doi: 10.1002/jbm.a.30069 15307157

[30] Schindelin J Arganda-Carreras I Frise E Kaynig V Longair M Pietzsch T. Fiji: an open-source platform for biological-image analysis. Nat Methods. (2012) 9:676–82. doi: 10.1038/nmeth.2019 PMC3855844 22743772

[31] Hamidzadeh K Belew AT El-Sayed NM Mosser DM. The transition of M-CSF–derived human macrophages to a growth-promoting phenotype. Blood Adv. (2020) 4:5460–72. doi: 10.1182/bloodadvances.2020002683 PMC7656919 33166408

[32] Hamilton JA. Colony-stimulating factors in inflammation and autoimmunity. Nat Rev Immunol. (2008) 8:533–44. doi: 10.1038/nri2356 18551128

[33] Ushach I Zlotnik A. Biological role of granulocyte macrophage colony-stimulating factor (GM-CSF) and macrophage colony-stimulating factor (M-CSF) on cells of the myeloid lineage. J Leukoc Biol. (2016) 100:481–9. doi: 10.1189/jlb.3RU0316-144R PMC4982611 27354413

[34] Becher B Tugues S Greter M. GM-CSF: from growth factor to central mediator of tissue inflammation. Immunity. (2016) 45:963–73. doi: 10.1016/j.immuni.2016.10.026 27851925

[35] Kyriakides TR Foster MJ Keeney GE Tsai A Giachelli CM Clark-Lewis I. The CC chemokine ligand, CCL2/MCP1, participates in macrophage fusion and foreign body giant cell formation. Am J Pathol. (2004) 165:2157–66. doi: 10.1016/S0002-9440(10)63265-8 PMC1618731 15579457

[36] ten Harkel B Schoenmaker T Picavet DI Davison NL de Vries TJ Everts V. The foreign body giant cell cannot resorb bone, but dissolves hydroxyapatite like osteoclasts. PloS One. (2015) 10:e0139564. doi: 10.1371/journal.pone.0139564 26426806 PMC4591016

[37] McWhorter FY Wang T Nguyen P Chung T Liu WF. Modulation of macrophage phenotype by cell shape. Proc Natl Acad Sci U S A. (2013) 110:17253–8. doi: 10.1073/pnas.1308887110 PMC3808615 24101477

[38] Kao WJ McNally AK Hiltner A Anderson JM. Role for interleukin-4 in foreign-body giant cell formation on a poly(etherurethane urea) in vivo. J BioMed Mater Res. (1995) 29:1267–75. doi: 10.1002/jbm.820291014 8557729

[39] Junttila IS. Tuning the cytokine responses: An update on interleukin (IL)-4 and IL-13 receptor complexes. Front Immunol. (2018) 9:888. doi: 10.3389/fimmu.2018.00888 29930549 PMC6001902

[40] Yang J Jao B McNally AK Anderson JM. *In vivo* quantitative and qualitative assessment of foreign body giant cell formation on biomaterials in mice deficient in natural killer lymphocyte subsets, mast cells, or the interleukin-4 receptorα and in severe combined immunodeficient mice. J BioMed Mater Res A. (2014) 102:2017–23. doi: 10.1002/jbm.a.35152 24616384

[41] Jones JA Chang DT Meyerson H Colton E Kwon IK Matsuda T. Proteomic analysis and quantification of cytokines and chemokines from biomaterial surface-adherent macrophages and foreign body giant cells. J BioMed Mater Res A. (2007) 83:585–96. doi: 10.1002/jbm.a.31221 17503526

[42] McNally AK Jones JA Macewan SR Colton E Anderson JM. Vitronectin is a critical protein adhesion substrate for IL-4-induced foreign body giant cell formation. J BioMed Mater Res A. (2008) 86:535–43. doi: 10.1002/jbm.a.31658 PMC4227597 17994558

[43] Collier TO Anderson JM. Protein and surface effects on monocyte and macrophage adhesion, maturation, and survival. J BioMed Mater Res. (2002) 60:487–96. doi: 10.1002/jbm.10043 11920674

[44] Helming L Gordon S. Macrophage fusion induced by IL-4 alternative activation is a multistage process involving multiple target molecules. Eur J Immunol. (2007) 37:33–42. doi: 10.1002/eji.200636788 17154265

[45] Zhou H Xue Y Dong L Wang C. Biomaterial-based physical regulation of macrophage behaviour. J Mater Chem B Mater Biol Med. (2021) 9:3608–21. doi: 10.1039/D1TB00107H 33908577

[46] Piccinini AM Midwood KS. DAMPening inflammation by modulating TLR signalling. Mediators Inflammation. (2010) 2010:672395. doi: 10.1155/2010/672395 PMC2913853 20706656

[47] Swartzlander MD Barnes CA Blakney AK Kaar JL Kyriakides TR Bryant SJ. Linking the foreign body response and protein adsorption to PEG-based hydrogels using proteomics. Biomaterials. (2015) 41:26–36. doi: 10.1016/j.biomaterials.2014.11.026 25522962 PMC4629245

[48] McKiel LA Fitzpatrick LE. Toll-like receptor 2-dependent NF-κB/AP-1 activation by damage-associated molecular patterns adsorbed on polymeric surfaces. ACS Biomater Sci Eng. (2018) 4:3792–801. doi: 10.1021/acsbiomaterials.8b00613 33429600

[49] Nandi A Dey S Biswas J Jaiswal P Naaz S Yasmin T. Differential induction of inflammatory cytokines and reactive oxygen species in murine peritoneal macrophages and resident fresh bone marrow cells by acute staphylococcus aureus infection: contribution of toll-like receptor 2 (TLR2). Inflammation. (2015) 38:224–44. doi: 10.1007/s10753-014-0026-8 25266881

[50] Anderson JM Jiang S. Implications of the Acute and Chronic Inflammatory Response and the Foreign Body Reaction to the Immune Response of Implanted Biomaterials. In: Corradetti B, editor. The Immune Response to Implanted Materials and Devices: The Impact of the Immune System on the Success of an Implant. Springer International Publishing, Cham (2017). p. 15–36.

[51] Zhan Y Lew AM Chopin M. The pleiotropic effects of the GM-CSF rheostat on myeloid cell differentiation and function: more than a numbers game. Front Immunol. (2019) 10:2679. doi: 10.3389/fimmu.2019.02679 31803190 PMC6873328

[52] Cechim G Chies JAB. *In vitro* generation of human monocyte-derived dendritic cells methodological aspects in a comprehensive review. Acad Bras Cienc. (2019) 91:e20190310. doi: 10.1590/0001-3765201920190310

[53] Ahn JS Agrawal B. IL-4 is more effective than IL-13 for in *vitro* differentiation of dendritic cells from peripheral blood mononuclear cells. Int Immunol. (2005) 17:1337–46. doi: 10.1093/intimm/dxh312 16141241

[54] Çaykara T Sande MG Azoia N Rodrigues LR Silva CJ. Exploring the potential of polyethylene terephthalate in the design of antibacterial surfaces. Med Microbiol Immunol. (2020) 209:363–72. doi: 10.1007/s00430-020-00660-8 PMC7248016 32037497

[55] Amri A Chevallier P Guay-Bégin A-A Bilem I Gauvin G Alamdari H. Polyethylene terephthalate textile heart valve: How poly(ethylene glycol) grafting limits fibrosis. J BioMed Mater Res B Appl Biomater. (2022) 110:2110–20. doi: 10.1002/jbm.b.35065 35420261

[56] ECRI. Medical device material performance study: polyethylene terephthalate (PET) safety profile. ECRI. (2020).

[57] Hiasa M Abe M Nakano A Oda A Amou H Kido S. GM-CSF and IL-4 induce dendritic cell differentiation and disrupt osteoclastogenesis through M-CSF receptor shedding by up-regulation of TNF-alpha converting enzyme (TACE). Blood. (2009) 114:4517–26. doi: 10.1182/blood-2009-04-215020 19762488

[58] Doloff JC Veiseh O Vegas AJ Tam HH Farah S Ma M. Colony stimulating factor-1 receptor is a central component of the foreign body response to biomaterial implants in rodents and non-human primates. Nat Mater. (2017) 16:671–80. doi: 10.1038/nmat4866 PMC5445003 28319612

[59] Xie J Qian J Wang S Freeman ME 3rd Epstein J Yi Q. Novel and detrimental effects of lipopolysaccharide on in *vitro* generation of immature dendritic cells: involvement of mitogen-activated protein kinase p38. J Immunol. (2003) 171:4792–800. doi: 10.4049/jimmunol.171.9.4792 14568957

[60] Czimmerer Z Halasz L Daniel B Varga Z Bene K Domokos A. The epigenetic state of IL-4-polarized macrophages enables inflammatory cistromic expansion and extended synergistic response to TLR ligands. Immunity. (2022) 55:2006–2026.e6. doi: 10.1016/j.immuni.2022.10.004 36323312 PMC9649892

[61] Boissy P Machuca I Pfaff M Ficheux D Jurdic P. Aggregation of mononucleated precursors triggers cell surface expression of alphavbeta3 integrin, essential to formation of osteoclast-like multinucleated cells. J Cell Sci. (1998) 111:2563–74. doi: 10.1242/jcs.111.17.2563 9701555

[62] Sanjabi S Zenewicz LA Kamanaka M Flavell RA. Anti-inflammatory and pro-inflammatory roles of TGF-beta, IL-10, and IL-22 in immunity and autoimmunity. Curr Opin Pharmacol. (2009) 9:447–53. doi: 10.1016/j.coph.2009.04.008 PMC2755239 19481975

[63] Fabre T Barron AMS Christensen SM Asano S Bound K Lech MP. Identification of a broadly fibrogenic macrophage subset induced by type 3 inflammation. Sci Immunol. (2023) 8:eadd8945. doi: 10.1126/sciimmunol.add8945 37027478

[64] Ullm F Riedl P MaChado de Amorim A Patzschke A Weiß R Hauschildt S. 3D scaffold-based macrophage fibroblast coculture model reveals IL-10 dependence of wound resolution phase. Adv Biosyst. (2020) 4:e1900220. doi: 10.1002/adbi.201900220 32293120

[65] Jannasch M Gaetzner S Groeber F Weigel T Walles H Schmitz T. An in *vitro* model mimics the contact of biomaterials to blood components and the reaction of surrounding soft tissue. Acta Biomater. (2019) 89:227–41. doi: 10.1016/j.actbio.2019.03.029 30880238

[66] Zhou G Liedmann A Chatterjee C Groth T. *In vitro* study of the host responses to model biomaterials *via* a fibroblast/macrophage co-culture system. Biomater Sci. (2016) 5:141–52. doi: 10.1039/C6BM00247A 27909707

[67] Pierce GF Mustoe TA Lingelbach J Masakowski VR Griffin GL Senior RM. Platelet-derived growth factor and transforming growth factor-beta enhance tissue repair activities by unique mechanisms. J Cell Biol. (1989) 109:429–40. doi: 10.1083/jcb.109.1.429 PMC2115493 2745556

[68] Li R Feng D Han S Zhai X Yu X Fu Y. Macrophages and fibroblasts in foreign body reactions: How mechanical cues drive cell functions? Mater Today Bio. (2023) 22:100783. doi: 10.1016/j.mtbio.2023.100783 PMC10494263 37701130

[69] Ward NA Hanley S Tarpey R Schreiber LHJ O’Dwyer J Roche ET. Intermittent actuation attenuates fibrotic behaviour of myofibroblasts. Acta Biomater. (2023) 173:80–92. doi: 10.1016/j.actbio.2023.11.017 37967693

[70] Bhattacharyya S Midwood KS Varga J. Tenascin-C in fibrosis in multiple organs: Translational implications. Semin Cell Dev Biol. (2022) 128:130–6. doi: 10.1016/j.semcdb.2022.03.019 PMC10119770 35400564

[71] McKiel LA Ballantyne LL Negri GL Woodhouse KA Fitzpatrick LE. MyD88-dependent Toll-like receptor 2 signaling modulates macrophage activation on lysate-adsorbed Teflon^TM^ AF surfaces in an in *vitro* biomaterial host response model. Front Immunol. (2023) 14:1232586. doi: 10.3389/fimmu.2023.1232586 37691934 PMC10491479

