# Supplementary material for: Cellular and microenvironmental cues that promote macrophage fusion and foreign body response
Source: Front Immunol. 2024 Jul 5;15:1411872. doi: 10.3389/fimmu.2024.1411872 (PMC11257916; doi:10.3389/fimmu.2024.1411872)
Supplement: Supplementary file 1 [file DataSheet_1.pdf]

Supplementary method information

Water contact angle

The sessile drop technique was employed to calculate the static water contact angle on TCPS and PET using a contact angle goniometer (KSV CAM 200). One droplet was deposited per sample area with five different sampling areas measured for TCPS and PET. Greyscale images were captured at one-second intervals over a 20-second period with the first two frames discarded for all repeats. Data is reported as mean water contact angle at t=20 seconds ± standard deviation (SD).

Atomic force microscopy (AFM)

Prior to surface roughness measurements, material surfaces were cleaned with lint-free wipes to remove fine dust and debris. Surface roughness was characterised by AFM using the Bruker Dimension Icon with a RTESPA-150 probe (tip radius 8 nm). The peakforce setpoint was set to 15 nN and areas of 5 μm^2 (n=3) were scanned at a frequency of 1 Hz in tapping mode. Data were analysed using Gwyddion[95] software (version 2.61) in which data were first processed by removal of polynomial background followed by row alignment using the median method. The roughness of the 3D surface (R_a) was calculated within the software and data reported as the mean ± SD from triplicate repeats. 3D images representing the surface topography of TCPS and PET were created in Gwyddion.

The stiffness of the material substrates was also determined by AFM using the Bruker Dimension Icon. An RTESPA-300 probe (tip radius 8 nm) with a spring constant calibrated to be 17.6 N/m was indented into sample surfaces at 20 points (4x5) using a step size of 200 nm. The data were analysed in Bruker Nanoscope Analysis (version 1.90) wherein the baseline was corrected followed by indentation analysis using the linearised fit method with the Hertzian model. The Young's Modulus was calculated within the software and reported as the mean ± SD of twenty sample points.

Fourier transform infrared (FTIR) spectroscopy

Functional groups of PET and TCPS were identified with the Agilent Cary 630 FTIR spectrometer using the Diamond ATR attachment. Raw data was exported into GraphPad Prism (version 9.4.1) for figure construction and analysis.

Supplementary figures

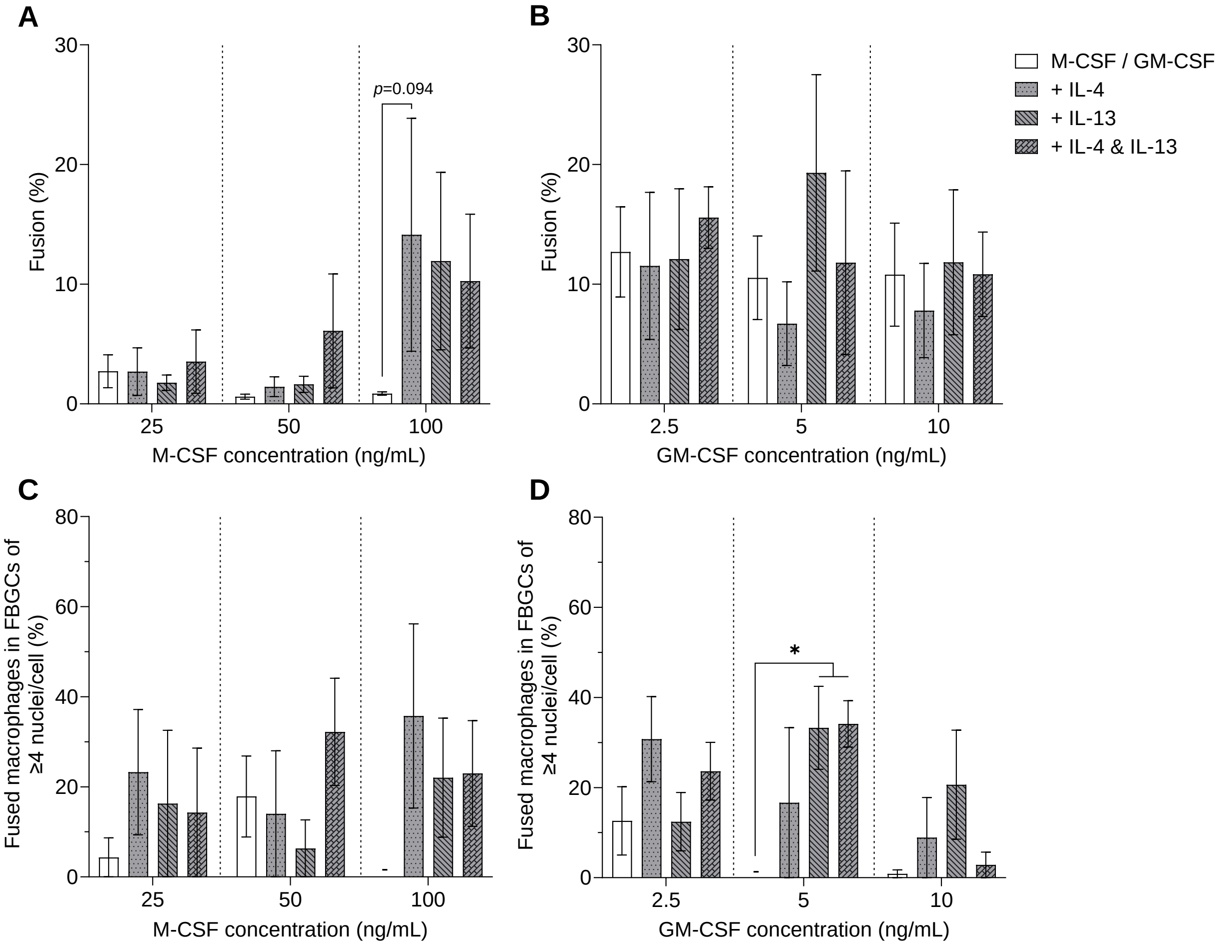

Fig S1. Effect of M-CSF and GM-CSF concentration on macrophage fusion and size of FBGCs. Percentage of macrophage fusion in response to **(a)** M-CSF (25-100 ng/mL) and **(b)** GM-CSF (2.5-10 ng/mL). Percentage of fused macrophages that formed FBGCs containing ≥4 nuclei/cell when cultured in **(c)** M-CSF (25-100 ng/mL) and **(d)** GM-CSF (2.5-10 ng/mL). Macrophages were cultured in the absence or presence of IL-4 (10 ng/mL), IL-13 (10 ng/mL or both IL-4 (5 ng/mL) and IL-13 (5 ng/mL). Data are means ± SEM of three donors. Statistical significance was determined by two-way ANOVA with the Dunnett multiple comparison test, wherein each experimental condition containing interleukins was compared to its respective control condition consisting of M-CSF or GM-CSF without interleukins (d: $F_{6,20}$=1.87, * p<0.05).

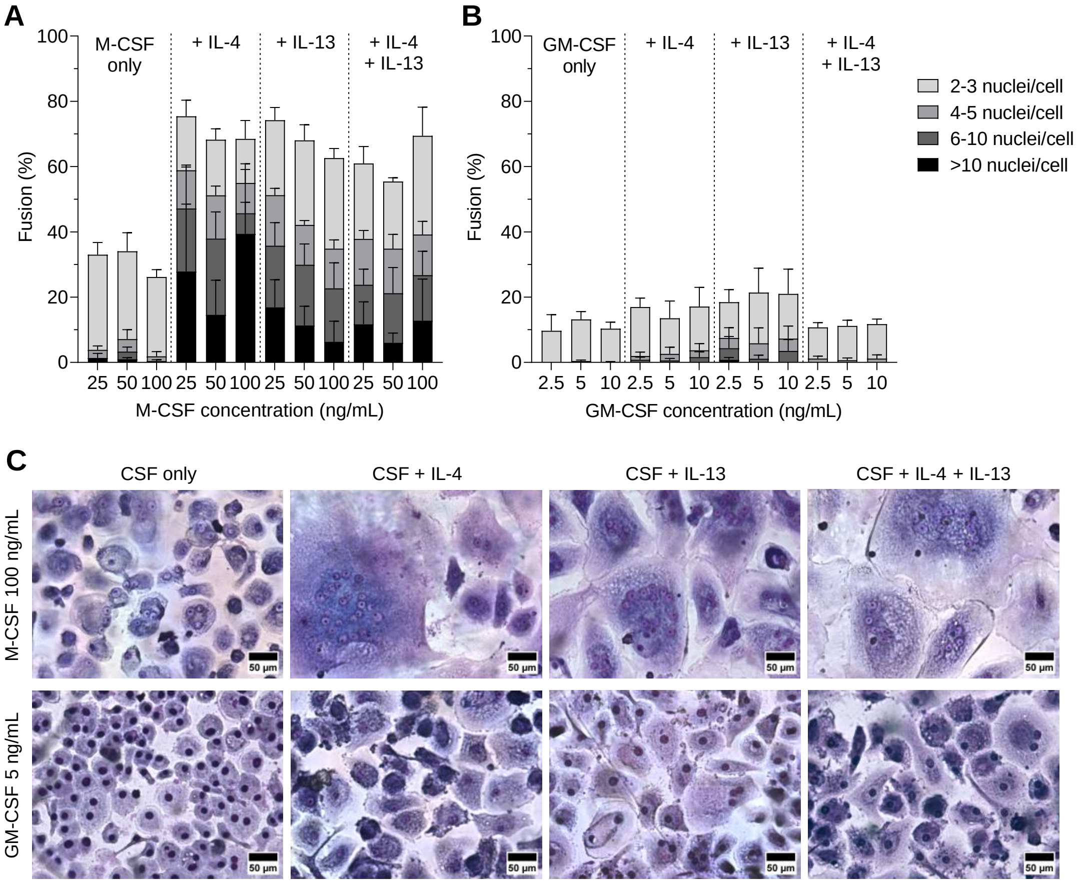

Fig S2. Effect of M-CSF and GM-CSF concentration on FBGC formation on PET. Percentage of macrophage fusion on PET in response to **(a)** M-CSF (25-100 ng/mL) and **(b)** GM-CSF (2.5-10 ng/mL) with stacked bars showing the percentage of nuclei within FBGC containing a total of 2 to 3 nuclei/cell, 4 to 5 nuclei/cell, 6 to 10 nuclei/cell and/or >10 nuclei/cell. Data are means ± SEM of three donors. **(c)** Macrophages and FBGCs were stained with May-Grünwald and Giemsa and imaged after 28 days of culture in different cytokine cocktails. Images are representative of three independent experiments, each with a different donor. Scale bar, 50 µm.

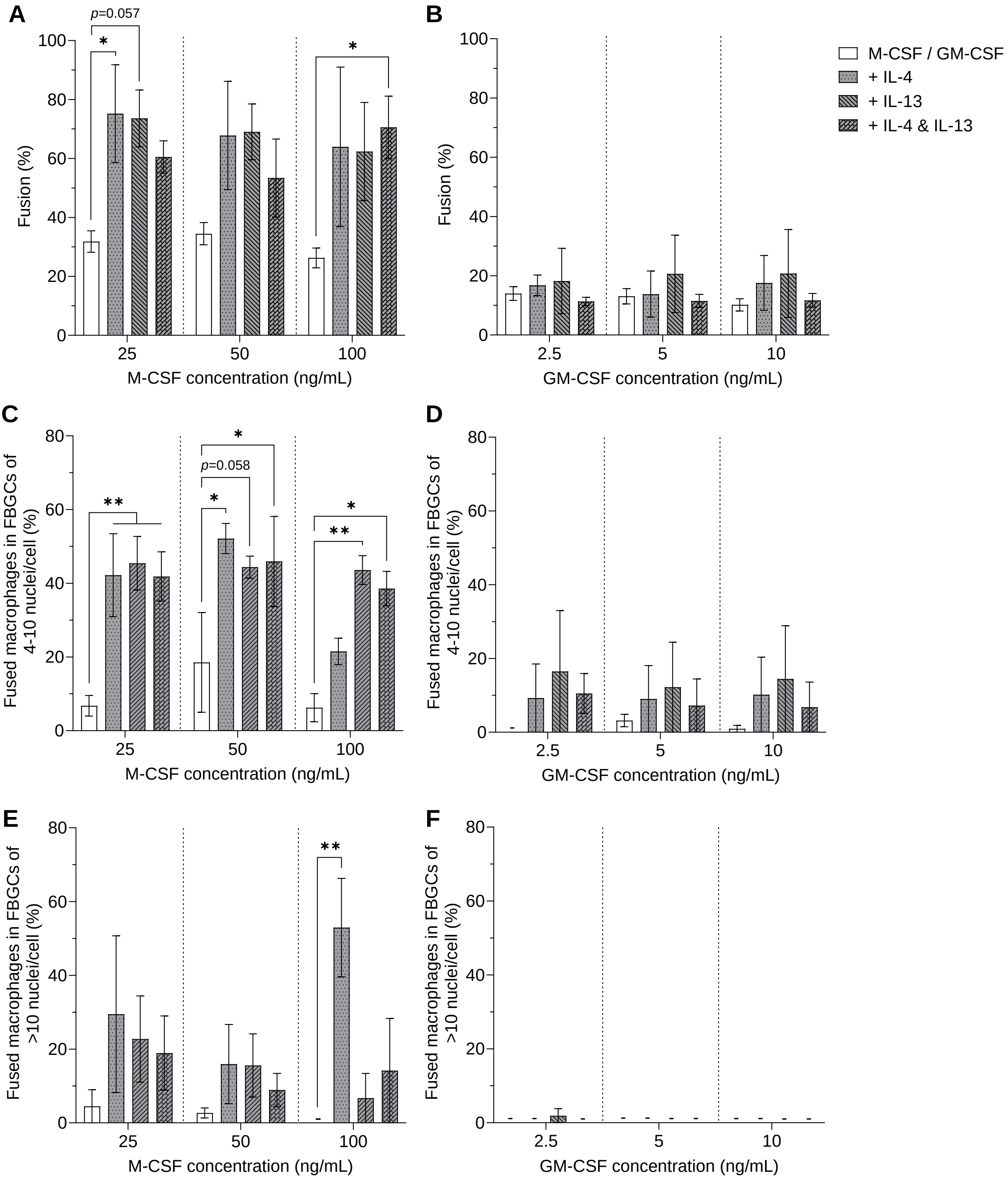

Fig S3. Effect of M-CSF and GM-CSF concentration on macrophage fusion and size of FBGCs on PET. Percentage of macrophage fusion on PET in response to **(a)** M-CSF (25-100 ng/mL) and **(b)** GM-CSF (2.5-10 ng/mL). Percentage of fused macrophages that formed FBGCs containing 4 to 10 nuclei/cell and >10 nuclei/cell when cultured on PET and with **(c, e)** M-CSF (25-100 ng/mL) and **(d, f)** GM-CSF (2.5-10 ng/mL). Data are means ± SEM of three donors. Statistical significance was determined by two-way ANOVA with the Dunnett multiple comparison test, wherein each experimental condition containing interleukins was compared to the respective control condition of M-CSF or GM-CSF without interleukins (a: $F_{6,23}$=0.30; c: $F_{6,23}$=0.72, e: $F_{6,23}$=0.94; * p<0.05, ** p<0.01).

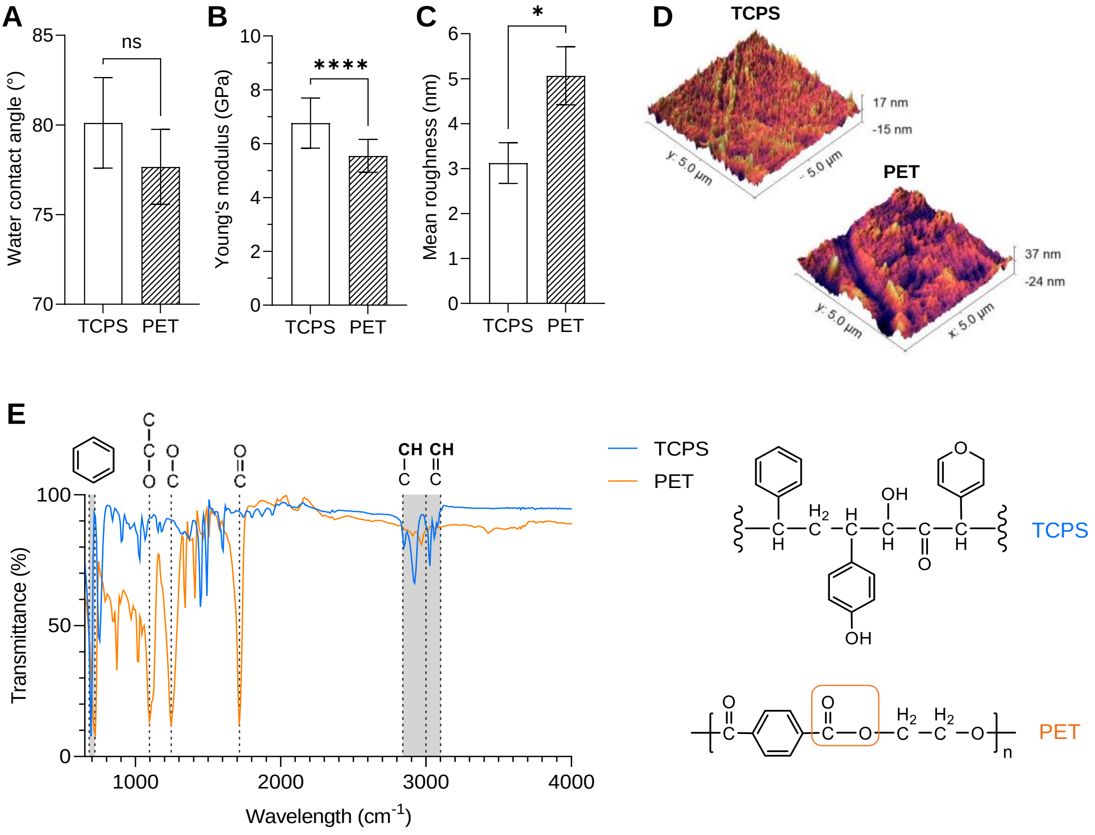

Fig S4. Material characterisation of TCPS and PET. (a) Water contact angle of TCPS and PET surfaces. Data is the mean ± SD of five droplets, each deposited onto a different region of a material sample. No statistical significance determined by an unpaired t-test (t=1.68, df=8). **(b)** Young's modulus determined by atomic force microscopy (AFM) nanoindentation on TCPS and PET surfaces. Data is the mean ± SD of twenty areas of a material sample. Statistical significance was determined by an unpaired t-test (t=4.91, df=38, **** p<0.001). **(c)** Average roughness (R_a) of TCPS and PET surfaces measured by AFM over a 5 µm^2 area. Data is the mean ± SD of three areas. Statistical significance was determined by an unpaired t-test (t=4.27, df=4, * p<0.05). **(d)** 3D topographical projections of TCPS and PET surfaces generated from AFM roughness data. **(e)** Chemical functional groups identified by fourier transform infrared (FTIR) spectroscopy. Each key signal identified is labelled with the corresponding chemical structure. The repeating chemical units of the TCPS and PET polymers are depicted next to the FTIR spectrum with the PET ester group highlighted.

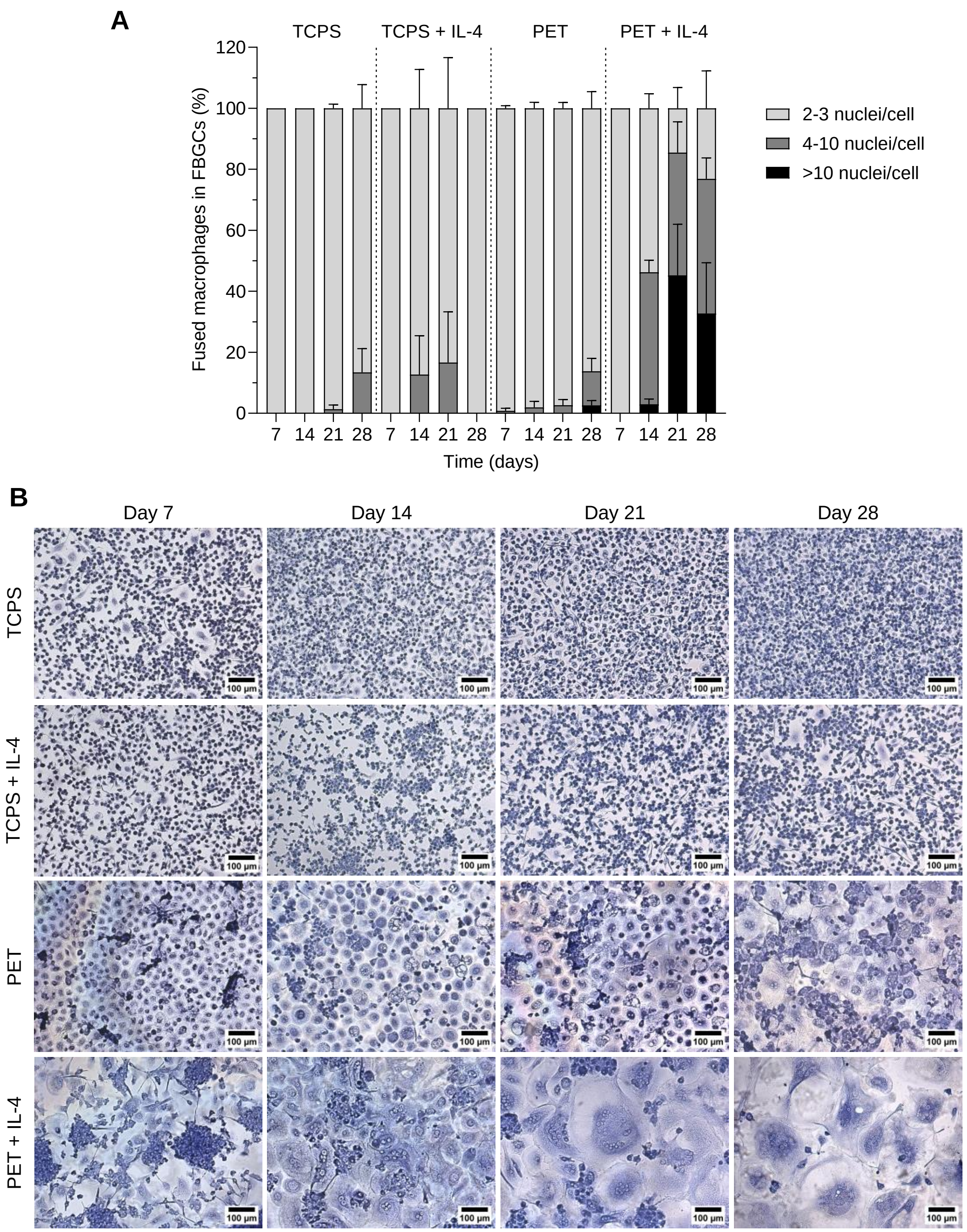

Fig S5. Timescale of FBGC formation in response to different experimental conditions. (a) Percentage of fused macrophages that contained either 2 to 3 nuclei/cell, 4 to 10 nuclei/cell and >10 nuclei/cell at weekly intervals when macrophages were cultured on TCPS or PET and differentiated by M-CSF (50 ng/mL) with or without IL-4 (10 ng/mL) polarisation. Data are means ± SEM of four biological donors. **(b)** Representative images of M-CSF-differentiated macrophages and FBGCs stained with May-Grünwald and Giemsa at weekly intervals when cultured on TCPS or PET in the presence and absence of IL-4. Scale bar, 100 µm.

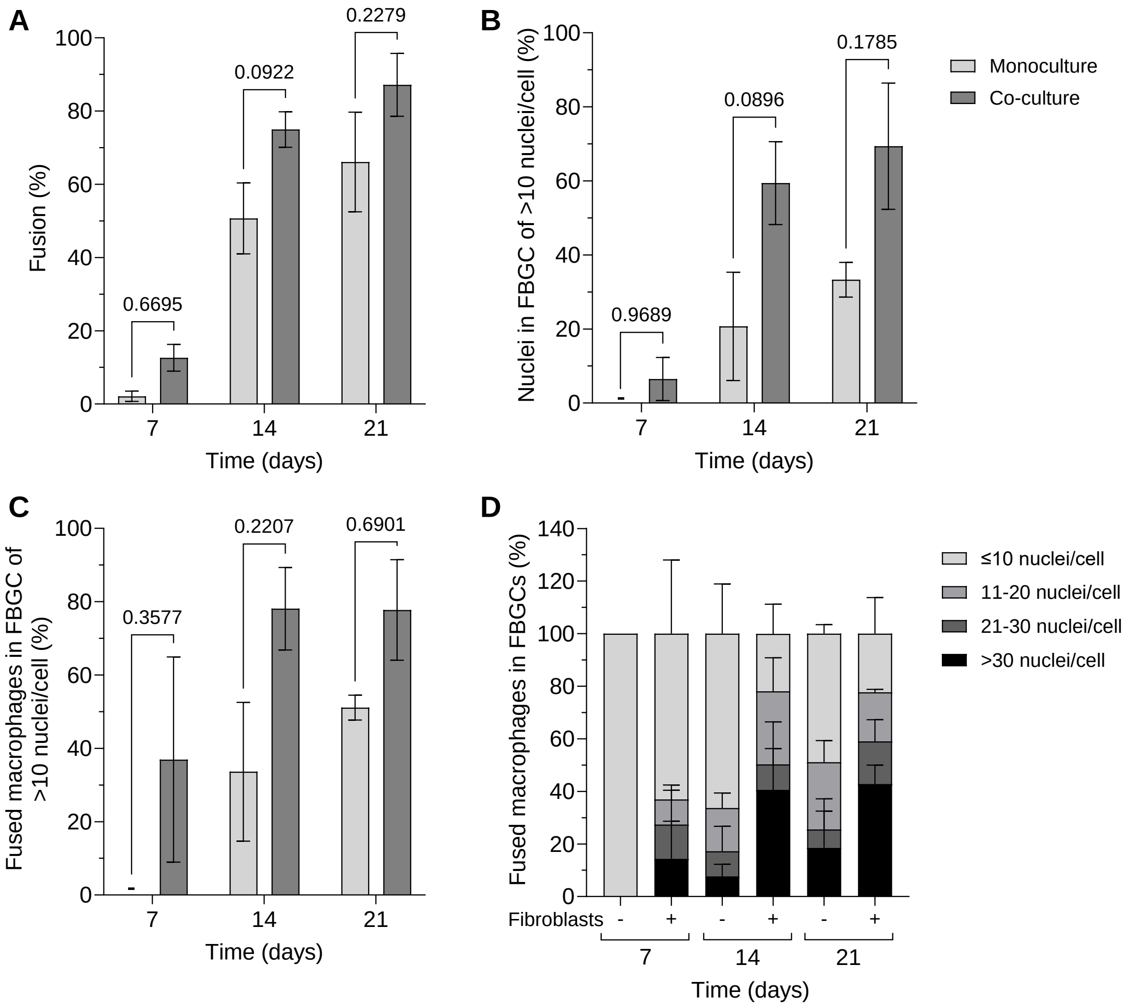

Fig S6. Effect of fibroblasts on macrophage fusion and size of FBGCs in macrophage-fibroblast indirect co-cultures. (a) Percentage of total macrophage fusion, **(b)** percentage of macrophages that fused into FBGC of >10 nuclei/cell and **(c)** percentage of fused macrophages in FBGC of >10 nuclei/cell at weekly intervals. Macrophages were differentiated by M-CSF (50 ng/mL) and polarised by IL-4 (10 ng/mL). Data are the means ± SEM of three biological donors and no statistical significance was determined by two-way ANOVA with the Šídák multiple comparison test. **(d)** Data represented as stacked bars showing the percentage of fused macrophages that formed FBGCs containing ≤10 nuclei/cell, 11 to 20 nuclei/cell, 21 to 30 nuclei/cell and/or >30 nuclei/cell.

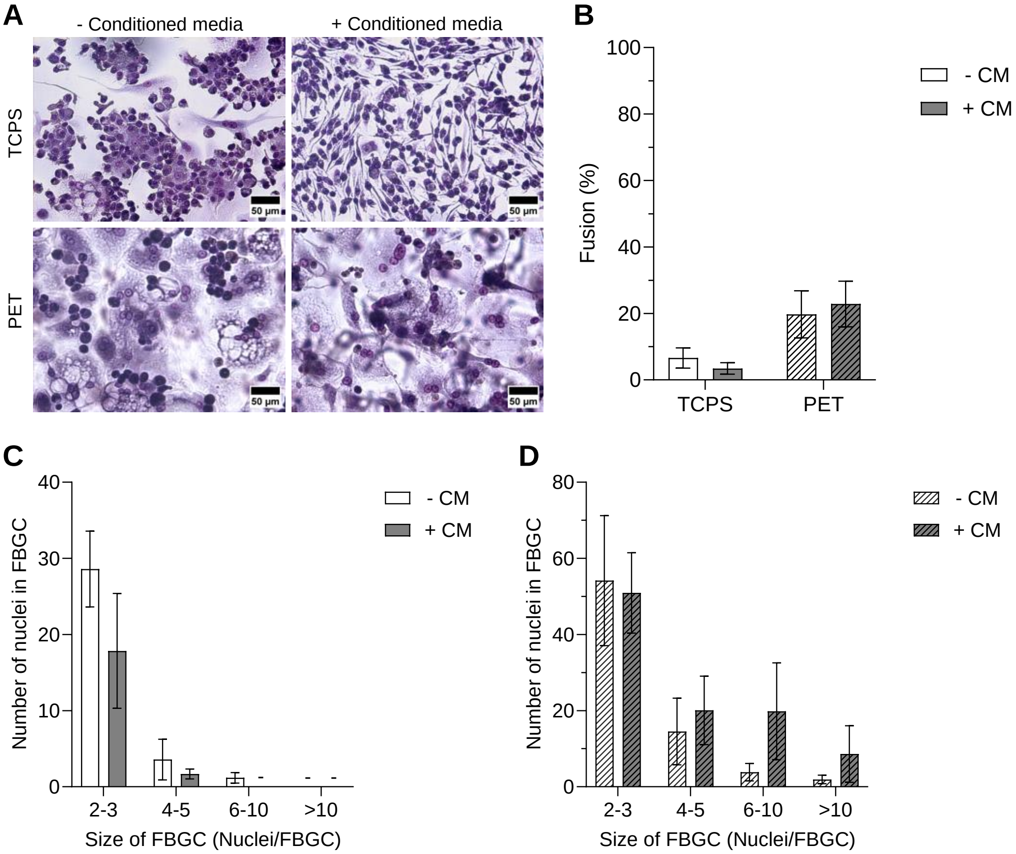

Fig S7. Effect of fibroblast-conditioned medium on macrophages cultured in non-fusogenic conditions. (a) Representative images of May-Grünwald and Giemsa-stained macrophages and FBGCs when cultured on TCPS and PET and differentiated by M-CSF (50 ng/mL) in the presence and absence of fibroblast-conditioned medium (CM) for 21 days. Scale bar, 50 μm. **(b)** Percentage of macrophage fusion induced on TCPS and PET by the addition of fibroblast-CM. Number of nuclei counted in FBGC containing either 2 to 3 nuclei/cell, 4 to 5 nuclei/cell, 6 to 10 nuclei/cell and >10 nuclei/cell when macrophages were cultured on **(c)** TCPS and **(d)** PET. Data are the means ± SEM of four biological donors with no statistical significance determined by two-way ANOVA with the Šídák multiple comparison test.

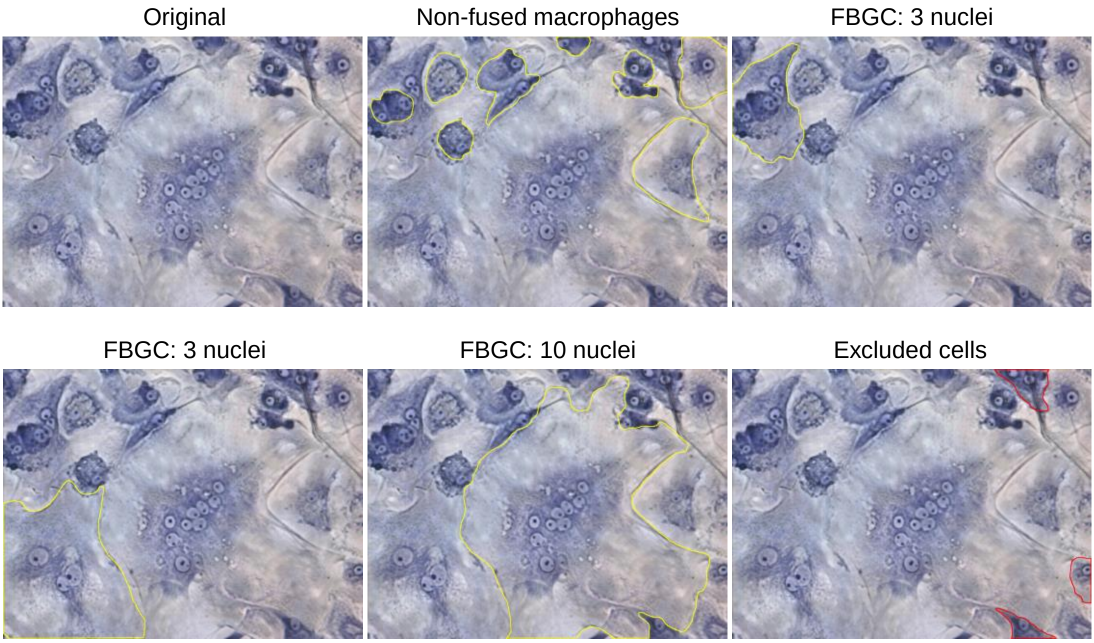

Fig S8. Example of counting nuclei in FBGCs. Non-fused macrophages containing a single nucleus per cell and multinucleated cells (3 nuclei/cell and 10 nuclei/cell) are outlined in yellow and included in data measurements. Cells at the edge of the image that are partially obscured are outlined in red and excluded from measurements.
